# Integrative RNA sequencing uncovers the miRNA–mRNA landscape of water-deficit response in *Setaria italica*

**DOI:** 10.1007/s00425-026-05117-5

**Published:** 2026-08-10

**Authors:** Juliana M. Rodrigues, Vitor G. B. Theodoro, Gabriel G. Carvalho, Igor Cesarino, Leandro F. de Oliveira, Nathalia de Setta

**Affiliations:** 1 Centro de Ciências Naturais E Humanas (CCNH), Universidade Federal Do ABC (UFABC), São Bernardo Do Campo, SP Brazil; 2 LigninLab, Department of Botany, Institute of Biosciences, University of São Paulo, São Paulo, SP Brazil

**Keywords:** Abiotic stress, Degradome sequencing, Gene expression, Monocotyledons, Post-transcriptional regulation, Transcriptome

## Abstract

**Main conclusion:**

**In miRNA–mRNA regulatory modules of** ***Setaria italica*** **, the candidate novel miRNAs Sit-miR74-N/APX7 and Sit-miR132-N/PEPC involved in key pathways underlying water-deficit response have been detected. In the first module,** ***APX7*** **expression was directly associated with water-deficit intensity, whereas in the second module, PEPC activation occurred under moderate water deficit.**

**Abstract:**

Drought severely constrains plant growth and productivity, yet the underlying regulatory mechanisms remain incompletely understood. Here, we determined the miRNA-mediated post-transcriptional landscape during water deficit in the panicoid grass *Setaria italica*. Two independent water-deficit experiments combined with small RNA and degradome sequencing identified 159 miRNAs, including 135 candidate novel sequences, of which 20 were differentially expressed under contrasting watering regimes. In silico analysis predicted 1,612 miRNA–mRNA regulatory modules, representing a broad landscape of potential post-transcriptional regulation. Complementarily, degradome sequencing identified 19 miRNA–mRNA modules, several of which exhibited inverse expression patterns, particularly under severe water-deficit conditions. Functional annotation of target mRNAs uncovered multiple functional processes, including transcriptional regulation, photosynthesis, redox homeostasis, and solute transport. Additionally, transient expression assays validated selected miRNA–mRNA modules, confirming the interaction between candidate novel miRNAs and water deficit–related mRNA targets. These modules are involved in key water deficit–responsive pathways, including oxidative stress regulation (Sit-miR74-N/*APX7*), photosynthetic carbon metabolism (Sit-miR132-N/*PEPC*), and flavonoid biosynthetic pathways (Sit-miR39-N/*OMT*). Together, these findings reveal a complex regulatory network in which miRNAs contribute to the coordination of water-deficit responses in *S. italica*, with post-transcriptional regulation underlying plant responses to abiotic stress.

**Supplementary Information:**

The online version contains supplementary material available at 10.1007/s00425-026-05117-5.

## Introduction

Climate change is progressively intensifying environmental challenges, posing significant threats to multiple dimensions of human life, including food security, public health, and global economic stability. Among the abiotic stresses intensified by climate changes, drought stands out as one of the most restrictive factors for agricultural productivity (Kopecká et al. 2023). Prolonged or recurrent water deficits disrupt key developmental and reproductive processes in crop species, ultimately reducing both yield quantity and quality (Yadav et al. 2020). In Poaceae, water deficit triggers coordinated physiological and metabolic adjustments that include reductions in photosynthesis (Landi et al. 2017), activation of antioxidant defenses (Amoah et al. 2023), redirection of carbohydrate and amino acid metabolism toward osmotic balance and stress-related signaling (Lata et al. 2015), modifications in flowering time (Suguiyama et al. 2022), reproductive architecture (Kang and Futakuchi 2019), suppression of vegetative growth, and biomass accumulation (Sun et al. 2016). Given the increasing challenges posed by global climate change, unraveling the molecular mechanisms underlying water-deficit responses in plants is essential for the development of more resilient crops, ensuring global food security.

To orchestrate such stress responses, plants rely on complex genetic and epigenetic regulatory networks, including post-transcriptional gene expression control mediated by microRNAs (miRNAs). miRNAs guide sequence-specific cleavage or translational repression of target mRNAs, thereby fine-tuning gene expression and, consequently, protein abundance in response to environmental fluctuations (Abbas et al. 2022). miRNA–mRNA target modules have been associated with multiple plant biological processes, including development, cell wall biosynthesis, and stress responses. Several water deficit–responsive miRNAs have been reported to modulate genes associated with reactive oxygen species (ROS) detoxification, thereby contributing to cellular redox homeostasis under stress conditions and regulation of antioxidant enzymes, such as the miR398/*CSD* module described in maize and rice (Li et al. 2022) and the wheat miR1119/*MYC2* (Shamloo-Dashtpagerdi et al. 2023). In parallel, miRNA-mediated regulation of transcription factors constitutes an important layer of water-deficit response. For example, the tomato miR159/*MYB* module has been associated with osmoprotectant accumulation and improved water-deficit tolerance (López-Galiano et al. 2019), while the miR156/*SPL* module modulates metabolic and developmental responses to water deficit in maize and *Camellia sinensis* (Guo et al. 2017; Liu et al. 2019). Likewise, the maize miR164/*MYB* and miR164/*NAC* regulatory modules highlight the close interconnection between miRNA regulation, transcriptional control, and stress signaling pathways, as these transcription factors participate in water deficit–responsive signal transduction and ABA-mediated responses (Liu et al. 2019).

Significant gaps remain in the identification and functional characterization of miRNA–mRNA regulatory modules involved in water-deficit responses, particularly in grasses. *Setaria italica* (foxtail millet), a C4 panicoid grass domesticated from *Setaria viridis* (de Wet et al. 1979) has gradually adapted to arid and semiarid environments (Doust et al. 2009) and has emerged as a robust model for investigating photosynthesis, water-deficit response, and bioenergy-related traits in Poaceae. Initial genome-wide studies in *S. italica* established foundational miRNA resources by identifying conserved and novel miRNAs, characterizing their expression across tissues, and validating selected miRNA–target interactions, although these studies were conducted under nonstress conditions (Yi et al. 2013; Han et al. 2014; Deng et al. 2022). Subsequent studies expanded this knowledge by identifying water deficit–responsive miRNAs under abiotic stress conditions, including PEG-induced osmotic stress and moderate water deficit, revealing candidate regulatory modules associated with stress-responsive pathways (Khan et al. 2014; Yi et al. 2015; Wang et al. 2016). Collectively, these studies suggest that water deficit–responsive miRNAs in *S. italica* primarily regulate gene expression through target mRNA cleavage, predominantly targeting transcription factors as well as genes involved in cellular homeostasis. However, these studies were limited either by the use of osmotic stress as a water-deficit proxy or by the evaluation of a single water-deficit intensity, which does not fully capture the dynamic nature of plant responses to progressive water limitation. Therefore, despite substantial progress, a comprehensive understanding of water deficit–responsive miRNA–mRNA regulatory networks in *S. italica*, particularly under progressive water-deficit gradients and supported by robust experimental validation of miRNA–target interactions, remains lacking.

In this context, comprehensive approaches that integrate mRNA sequencing (RNA-seq), small RNA sequencing (sRNA-seq), and degradome analyses may help elucidate how plants acclimate under water deficit. RNA-seq and sRNA-seq libraries were previously generated from leaves of *S. italica* subjected to a water-deficit gradient experiment (Suguiyama et al. 2022). In that study, the sRNA analysis focused on siRNAs mapping to gene body regions of mRNAs (CDSs, introns, and UTRs), regulatory regions upstream of transcription start sites, and transposable elements. Here, we extend this framework by integrating previously generated RNA-seq and sRNA-seq datasets with newly generated degradome libraries and extensive target predictions, enabling the characterization of miRNA structure and function as well as the identification of miRNA–mRNA regulatory modules associated with severe and moderate water-deficit responses in *S. italica*. Our results suggest that the regulatory landscape of *S. italica* under contrasting watering regimes involves miRNA–mRNA modules that contribute to the water-deficit response network. This network spans multiple functional processes, including transcriptional regulation, photosynthetic adjustment, redox homeostasis, and solute transport, which may contribute to the fine-tuning of target gene expression and the coordination of plant responses to water deficit.

## Materials and methods

### Plant material and water-deficit treatments

Two independent water-deficit gradient experiments were performed by our research group. The first experiment was performed between February and August 2016 (Suguiyama et al. 2022), and the second between May and November 2022, using the same experimental design and conditions. All procedures were conducted in a controlled-environment growth chamber set to 26 ± 2 °C, 50–60% relative humidity, a 16 h light/8 h dark photoperiod, and a photosynthetic photon flux density (PPFD) of 400–500 µmol m⁻^2^ s⁻^1^. *Setaria italica* cv. YUGU1 seeds were germinated in 50 mL cups containing a 3:1 mixture of commercial substrate (Flores e folhagens, Biomix, https://biomix.com.br/) and vermiculite and irrigated daily. After 14 days, 30 seedlings were transplanted individually into 5 L pots filled with the same substrate: vermiculite mixture, supplemented with 1 g of NPK 10:10:10 and 4 g of dolomitic limestone (MgCO₃ + CaCO₃). Immediately after transplantation, the substrate was brought to field capacity, and pots were weighed using a conventional scale. After 48 h, the pots were reweighed to determine the mean evapotranspiration volume. This value was then used to define three irrigation conditions: 100% treatment (200 mL), 30% treatment (60 mL), and 0% treatment (no watering). Each treatment consisted of 10 biological replicates (one plant per pot). Plants were watered every 2 days until senescence. Soil water content (SWC) was estimated by time-domain reflectometry (TDR) using an ML3 ThetaProbe Moisture Meter HH2 sensor (Delta-T Devices), calibrated for organic soil. In the first and second experiments, leaf sampling was performed when the SWC of the 0% treatment approached zero and plants were in the leaf development stage of phenological BBCH-scale (Zadoks et al. 1974), at 38 and 51 days after the onset of the water-deficit treatment, respectively. At these sampling points, the 9th and 11th leaves from the plant base were harvested, flash-frozen in liquid nitrogen, and either stored at − 80 °C for molecular analyses or immediately processed to determine leaf relative water content (RWC). Plants from the first experiment were used for RNA-seq and sRNA-seq analyses (Suguiyama et al. 2022), whereas plants from the second experiment were used for degradome sequencing.

RWC was determined from two segments excised from the midpoint of the 9th and 11th leaves from the plant base, using the following equation: RWC (%) = (FW − DW/TW − DW) × 100, where FW is the fresh weight measured immediately after sampling, TW the turgid weight after 96 h of immersion in distilled water, and DW the dry weight obtained after drying at 70 °C for 96 h (Weatherley 1950). Yield-related traits were evaluated based on vegetative and reproductive parameters. Vegetative growth was assessed by maximum plant height (measured from the lowest stem node to the apical node), total number of leaves, and shoot biomass. Reproductive traits included emergence of panicle (days after sowing), weight of panicle, panicle length, and panicle width. Statistical analyses of yield-related traits and RWC were performed using one way ANOVA (Tukey’s post-hoc test, *P* ≤ 0.05), after confirming data normality using the modified Shapiro–Wilk’s test (*P* ≤ 0.05). In addition, a two-way ANOVA was applied to assess the consistency between the two independent experiments. All analyses were conducted in R using the multcomp package (https://multcomp.r-forge.r-project.org/).

### RNA extraction and sequencing

Total RNA was extracted from 100 mg of frozen, ground leaf tissue using TRIzol reagent (Invitrogen), according to the manufacturer’s instructions, followed by lithium chloride purification. RNA integrity and concentration were measured with BioDrop Touch Duo spectrophotometer (BioDrop, Waterbeach-Cambridge, UK) and Eukaryote Total RNA Pico Series II LabChip Kit of Bioanalyzer 2100 platform (Agilent Technologies). Samples with RNA integrity number (RIN) ≥ 6 were retained for sequencing. For RNA-seq and sRNA-seq, two biological replicates were prepared for each water-deficit treatment (100%, 30%, and 0%), with each replicate consisting of pooled RNA from two plants (four plants per treatment). A total of six libraries were generated for RNA-seq (100 bp paired-end reads) and six for sRNA-seq (50 bp single-end reads) on the Illumina HiSeq 4000 platform (BGI, Shenzhen, China), following standard Illumina protocols. Degradome libraries were prepared from pooled RNA (20 µg) obtained from three plants per treatment (nine plants total), using protocols described by Addo-Quaye et al. (2009) and German et al. (2008). Single-end degradome (50 bp reads) sequencing was performed on the Illumina HiSeq 2500 platform (CD Genomics, Shirley, NY, USA), following standard Illumina protocols.

### miRNA identification and differential expression analysis

sRNA-seq libraries were processed using a previously published pipeline (de Oliveira et al. 2023). Adapters were removed using Trim Galore and reads of 18–25 nt were retained with Cutadapt (Martin 2011). Quality filtering was performed with FastQC, keeping reads with a Phred score ≥ 30. rRNA and tRNA reads were removed after alignment using the RNAcentral database (The RNAcentral Consortium 2024) and Bowtie tool (Langmead et al. 2009). The remaining reads were used for miRNA prediction. miRNAs were identified with miRDeep2 (Friedländer et al. 2012), with a significant *P*-value ≤ 0.05. When both 5p and 3p sequences were detected, the most abundant arm was designated as the mature miRNA. Additionally, we applied the previously proposed filtering criteria (Axtell and Meyers 2018): (1) each precursor must contain only one miRNA/miRNA* duplex; (2) the foldback region must not contain secondary stems or internal loops larger than five nucleotides that disrupt the miRNA/miRNA* duplex; (3) the precursor sequence must be shorter than 300 nt; (4) precision, calculated as the sum of reads corresponding to the miRNA, miRNA*, and their respective one-nucleotide variants divided by the total number of reads aligned to the precursor, must be greater than 75%; (5) reads mapping to the miRNA locus must be detected in at least two sRNA-seq libraries derived from different treatments; and (6) the mature miRNA sequence must be 20–24 nucleotides in length.

Annotation of conserved miRNAs was performed using BLASTn (blastn-short) against Viridiplantae database of miRBase v22 (Kozomara et al. 2019) and *S. italica* records from PmiREN 2.1 (Guo et al. 2025). miRNAs were classified into three categories: conserved, known, or novel. Conserved miRNAs were defined as sequences matching miRNA families previously reported in other plant species and cataloged in miRBase v22 database. miRNA sequences already deposited in miRBase retained their database-assigned names (Kozomara et al. 2019), whereas the remaining conserved sequences were named according to their corresponding miRNA family, followed by sequential Arabic numerals. Known miRNAs were defined as those previously annotated in *S. italica* and retained the names assigned in the PmiREN 2.1 database (Guo et al. 2025). Candidate novel miRNAs corresponded to newly predicted sequences with no prior annotation in plant miRNA databases and were named using sequential Arabic numerals followed by -N, denoting novel. As an additional step, mature sequences of miRNAs classified as novel were compared against mature miRNA sequences reported in five previously published *S. italica* miRNA studies (Yi et al. 2013, 2015; Khan et al. 2014; Wang et al. 2016; Deng et al. 2022). Sequence comparisons were performed using BLASTn-short on mature miRNA sequences, allowing a maximum of two mismatches (≥ 90% identity). miRNA abundance across libraries was obtained using the Quantifier miRDeep2 module (Friedländer et al. 2012). Differentially expressed miRNAs (DEMs) were identified with edgeR v4.6.3 (Robinson et al. 2009). miRNAs with < 5 CPM in at least two libraries were excluded, and pairwise differential expression analysis was performed using CPM-normalized counts. miRNAs with *P* ≤ 0.05 and |log₂FC|≥ 1 were considered differentially expressed. Data visualization was conducted in R (R Core Team 2025) using ggplot2 (Wickham 2016), ComplexHeatmap (Gu 2022), dplyr (Wickham et al. 2023), and pheatmap (Kolde 2025) packages.

### Differential expression, prediction, and functional annotation of mRNAs

The *S. italica* transcriptome was subjected to differential expression analysis and RT-qPCR validation as reported previously (Suguiyama et al. 2022). Adaptor trimming and removal of low-quality bases were performed with Trimmomatic v0.32 (Bolger et al. 2014) retaining only reads with Phred scores ≥ 33. Clean reads were indexed and mapped to the *S. italica* v2.2 reference genome (Bennetzen et al. 2012) using GMAP/GSNAP v.2017–08–15 (Wu et al. 2016) with parameters allowing up to five mismatches per read (− m 5) and enabling splice site detection (− N 1). Read quantification per coding sequence (CDS) was performed using htseq-count v.0.8.0 (Putri et al. 2022). Gene-level expression modeling followed a negative binomial framework implemented in edgeR v.4.6.3 (Robinson et al. 2009) and DESeq2 (Love et al. 2014). Genes were considered differentially expressed if they met the criteria: FDR-adjusted *P*-value ≤ 0.05 and |log₂FC|≥ 1 using both edgeR and DESeq2 tools.

Putative mRNA targets were predicted using psRNATarget (Dai et al. 2018) under the V2 scoring schema and default parameters, applying a maximum expectation score threshold of ≤ 3.0. Predictions were performed against the mapped transcripts in the 100%, 30%, and 0% treatments RNA-seq libraries (Suguiyama et al. 2022). Functional annotation of predicted targets was performed using MapMan4 categories as implemented by the Mercator4 tool (Bolger et al. 2021) and GO and KEGG terms using EggNOG-mapper (Cantalapiedra et al. 2021) combined with DIAMOND-based sequence similarity searches (Buchfink et al. 2021).

### Degradome-based identification of miRNA–mRNA modules

Raw degradome reads were trimmed using Fastp v.0.23.2 (Chen et al. 2018) to remove adapters and low-quality bases, and the resulting clean reads were used to identify miRNA-guided mRNA cleavage sites with the CleaveLand4 pipeline (Addo-Quaye et al. 2009), aligned against the *S. italica* v2.2 transcriptome. Only perfect-match alignments were retained. The resulting t-signatures were reverse-complemented and aligned to miRNAs, allowing up to four mismatches. Candidate cleavage events were considered when the cleavage position corresponded to the 10th or 11th nucleotide of the miRNA. miRNA–mRNA modules were classified as degradome-validated when the cleavage site met a significance threshold of *P* ≤ 0.05. Identified interactions were classified into the five standard degradome categories based on the abundance distribution of degradome reads across the transcript: (i) category 0 corresponds to transcripts in which the read abundance at the cleavage position represents the unique maximum on the transcript; (ii) category 1 corresponds to cases in which the read abundance at the cleavage site is equal to the maximum read abundance observed in the transcript, but the maximum occurs at more than one position; (iii) category 2 includes transcripts in which the read abundance at the cleavage site is lower than the maximum but higher than the average abundance for that transcript; (iv) category 3 corresponds to cases in which the read abundance at the cleavage site is equal to or lower than the average read abundance; and (v) category 4 represents transcripts with only a single degradome read detected at the predicted cleavage position.

### Experimental validation of miRNA–target interactions using a RUBY-based reporter system

Three miRNA–mRNA modules, Sit-miR74-N/Seita.1G192100 (*APX7*), Sit-miR132-N/Seita.4G175200 (*PEPC*), and Sit-miR39-N/Seita.8G120600 (*OMT*), were validated using a RUBY-based reporter system coupled with *Agrobacterium*-mediated transient expression in *Nicotiana benthamiana* (He et al. 2020). Vector construction followed the Phytobricks modular cloning framework using a Golden Gate strategy (Cai et al. 2020). Level 0 (Lv0) parts corresponding to miRNA precursors (pre-miRNA) and target sequences flanked by appropriate Phytobrick overhangs and BsaI sites were commercially synthesized into pUC57-Kan (BioLinker, Suppl. Table S1), whereas those of promoters, terminators, and coding sequences (i.e., eGFP and RUBY cassette) were obtained from the MoClo Plant Parts III: Transformation & Genome Engineering Kit (#1,000,000,236, AddGene, Watertown, MA, USA). Negative controls were generated by replacing the miRNA precursor with an eGFP sequence while maintaining identical regulatory elements. Target fragments encompassed the predicted miRNA binding site flanked by 8 bp upstream and downstream. Level 1 (Lv1) expression cassettes were assembled using standard Golden Gate reactions with BsaI (New England Biolabs, Ipswich, MA, USA) and transformed into *Escherichia coli* DH10B. Transformants were selected on media supplemented with streptomycin and vector-specific antibiotics (Suppl. Table S1). Constructs were validated by colony PCR and restriction enzyme digestion using HindIII and BamHI. Digestion reactions were performed at 37 °C for 60 min followed by enzyme inactivation at 80 °C for 20 min, and fragment profiles were confirmed by agarose gel electrophoresis. Level 2 (Lv2) binary vectors (pMIN-VS1) were assembled using PaqCI (New England Biolabs) and introduced into *A. tumefaciens* strain GV3101. Correct L2 assembly was verified by restriction digestion using HindIII and subsequently confirmed by Oxford Nanopore-based whole plasmid sequencing (Plasmidsaurus, San Francisco, CA, USA). *Agrobacterium* transformants were selected using rifampicin and gentamicin, with vector-specific antibiotics (Suppl. Table S1).

For transient expression assays, *Agrobacterium* cultures were grown and prepared under selective conditions as described above. Bacterial suspensions were adjusted to an optical density (OD600) of approximately 0.8 in the infiltration buffer (10 mM MgCl₂, 10 mM MES, and 500 μM acetosyringone) and incubated in the dark for 1–4 h prior to infiltration. Fully expanded leaves of 4–6-week-old *N. benthamiana* plants were infiltrated on the abaxial side using a needleless syringe. At 7 days post-infiltration, leaf discs (6 mm diameter) were punched from infiltrated regions. Samples were homogenized using a bead mill in 100% ethanol. After centrifugation, the supernatant was discarded and the pellet resuspended in water for pigment extraction. Absorbance was measured at 538 nm using a Synergy H1 microplate reader (BioTek, Winooski, VT, USA). Measurements were obtained from one plant per module, using three leaves. Each leaf was divided into two halves (control and construct), with two infiltration spots per half. Data normality and homogeneity of variances were evaluated using the Shapiro–Wilk (*P* ≤ 0.05) and F (*P* ≤ 0.05) tests, respectively. Statistical differences were assessed using Student’s t-test (α *P* ≤ 0.05). Statistical analyses were performed in R (R Core Team 2025), and data visualization was conducted using the ggplot2 package (Wickham 2016).

## Results

### Water deficit restricts vegetative growth and reproductive development in *Setaria italica*

**Fig. 1:**
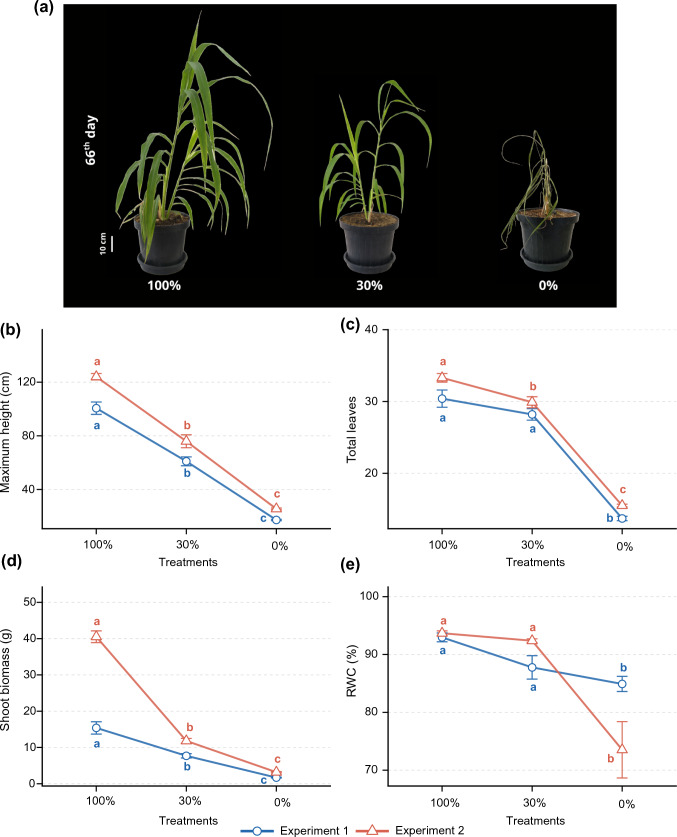
*Setaria italica* performance is impacted by water deficit treatment. **a** Representative image of *S. italica* plants from the second experiment, after 66 days of 100%, 30% and 0% water deficit treatments. **b** Maximum height. **c** Total number of leaves. **d** Shoot biomass. **e** Relative water content (RWC). Values represent the mean ± SE of measurements taken at the end of the experiment, 164 days after sowing. Different letters indicate statistically significant differences according to ANOVA (Tukey’s test, *P* ≤ 0.05, *n* = 10)

Two independent water-deficit experiments were conducted under identical growth conditions. Vegetative and reproductive traits of *S. italica* plants cultivated under full irrigation (100%), moderate (30%), and severe water deficit (0%) were evaluated to assess the impact of water restriction on plant development (Fig. 1). Most morphological and reproductive traits (i.e., weight, length, and width of panicles) exhibited consistent treatment responses across experiments, indicating strong reproducibility (Suppl. Table S2). However, shoot biomass and RWC showed significant experiment-by-treatment interactions, suggesting that although these traits responded to water deficit, their magnitude varied between experiments. Overall, the stable patterns observed across the remaining traits support the reliability of the treatment effects.

In both experiments, the 0% treatment severely limited vegetative growth, as evidenced by a 4.8–5.8-fold decrease in maximum height, a 9.1–12.7-fold decrease in shoot biomass, and an approximately 50% reduction in total leaf number (Fig. 1). Under 30% treatment, plants showed a milder response, with maximum height reduced 1.6-fold, shoot biomass reduced 2.0–3.4-fold, and leaf number reduced by 7–10%. RWC values showed a significant decrease in plants under the 0% treatment compared with those under the 100% and 30% treatments. For reproductive parameters, water-deficit treatments also had a pronounced impact (Table 1). Whereas plants under the 0% treatment senesced before reaching the reproductive stage, plants under the 30% treatment showed a delay in panicle emergence time of 28% in the first experiment and 5% in the second experiment relative to the 100% treatment. Additionally, they exhibited reductions in panicle weight (5.7 and 6.2 fold), panicle length (2.1 and 2.4 fold), and panicle width (2.0 and 1.4 fold) in the first and second experiments, respectively, compared with the 100% treatment. Altogether, these results showed that both moderate and severe water deficit negatively affects vegetative growth and reproductive development in *S. italica* in two independent experiments.

**Table 1:** Reproductive parameters (mean ± SE) of *S. italica* cultivated under three different water deficit treatments in two experiments

Trait	First experiment			Second experiment		
	100%	30%	0%	100%	30%	0%
Emergence of panicle (DAS)	97.1 ± 4.5^a^	124.6 ± 6.5^b^	−	117.76 ± 5.59^a^	123.94 ± 8.43^b^	−
Weight of panicle (g)	14.8 ± 2.2^a^	2.6 ± 0.3^b^	−	5.37 ± 0.72^a^	0.87 ± 0.15^b^	−
Panicle length (cm)	20.4 ± 1.4^a^	9.5 ± 0.5^b^	−	12.03 ± 0.68^a^	5.1 ± 0.33^b^	−
Panicle width (cm)	2.1 ± 0.2^a^	1.5 ± 0.0^b^	−	1.5 ± 0.06^a^	1.05 ± 0.08^b^	−

Different letters indicate statistically significant differences among treatments within the same experiment, as determined by Tukey’s test (*P* ≤ 0.05, *n* = 10). DAS: days after sowing

### Small RNA sequencing reveals conserved and candidate novel miRNAs in *Setaria italica* leaves under water deficit

To identify miRNA sequences associated with different intensities of water deficit in *S. italica*, sRNA-seq libraries were generated from leaves of plants subjected to 100%, 30%, and 0% irrigation treatments (Suguiyama et al. 2022). Each library yielded between 33 and 40 million raw reads, totaling approximately 223 million reads across the six libraries (Suppl. Table S3). After adapter removal, quality filtering, and size selection, 186 million reads were retained. The length distribution of these sRNAs showed a predominance of 24-nt reads, followed by 21- and 19-nts (Suppl. Fig. S1). Reads annotated as rRNA and tRNA represented 71.65% of the discarded fraction, while the remaining unmatched reads (28.35%), ranging from 2.86 to 11.00 million of reads per library, were used for miRNA prediction (Suppl. Table S4). The 5′ and 3′ nucleotide composition profiles of these unmatched sRNAs were also consistent across libraries, with the main differences observed for the 19-nt reads, which showed enrichment of G and U at the 5′ and 3′ ends, respectively (Suppl. Fig. S1).

To identify miRNAs in our dataset, unmatched sRNA reads were analyzed using miRDeep2 tool (Friedländer et al. 2012). miRNAs were classified into three categories: (i) conserved, when corresponding to sequences previously reported in other plant species in miRNA databases; (ii) known, when previously annotated in *S. italica* in miRNA databases, but not conserved across other species; (iii) novel, when consisting in newly candidate predicted sequences with no prior annotation in plant miRNA databases (Kozomara et al. 2019; Guo et al. 2025). A total of 159 mature miRNAs were identified, including 19 conserved, 5 known, and 135 candidate novel miRNAs (Suppl. Table S5). Additionally, novel miRNAs were used as queries in a BLAST based search against mature *S. italica* miRNAs reported in the literature (Yi et al. 2013, 2015; Khan et al. 2014; Wang et al. 2016; Deng et al. 2022). Among the 135 candidate novel miRNAs identified here, 16 had already been predicted in two of the evaluated datasets (Suppl. Table S5).

**Fig. 2:**
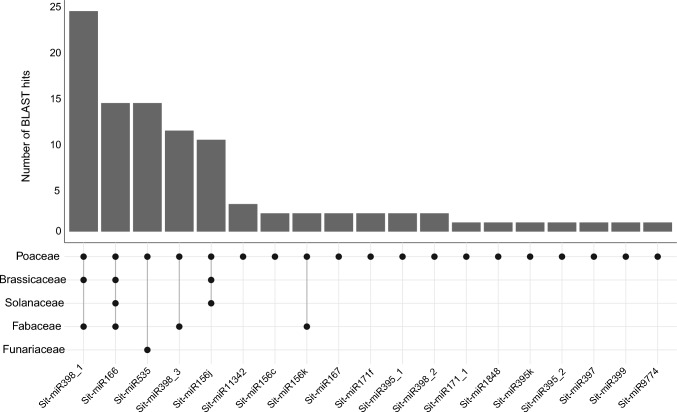
UpSet plot of the distribution of BLASTn-short hits for conserved *S. italica* miRNAs across plant families. The upper bar chart shows the number of hits identified for the mature miRNA sequences using BLASTn (blastn-short) tool against the Viridiplantae database of miRBase v22, with an e-value ≤ 0.001. The lower dot plot indicates the plant families in which homologous sequences were detected

**Fig. 3:**
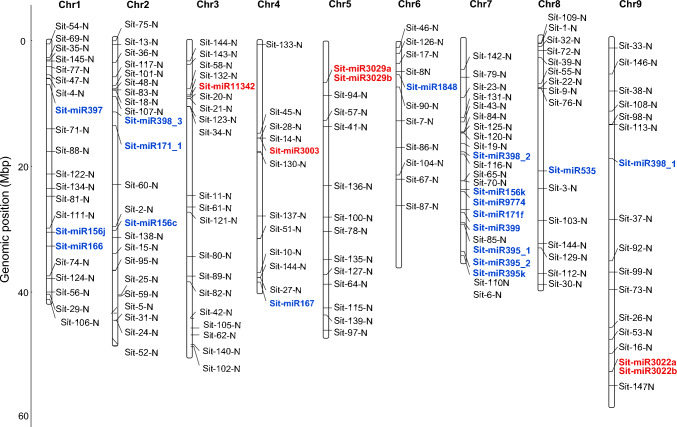
Physical map of *S. italica MIR* genes identified in this study. Genomic positions (Mbp) are shown on the left, and miRNA IDs are displayed on the right of chromosomes. Conserved, known and novel miRNAs are shown in blue, red, and black, respectively. Two miRNAs, Sit-miR128-N and Sit-miR118-N, mapped to scaffold_14 and scaffold_69, respectively, are not displayed in the physical map

The conserved miRNAs showed homology with miRNAs from at least one species within the Poaceae family, consistent with the phylogenetic position of *S. italica* (Fig. 2). Additional matches were observed in species from Brassicaceae, Fabaceae, and Solanaceae families. The most broadly distributed conserved miRNAs were Sit-miR398_1, Sit-miR166, Sit-miR156j, Sit-miR156c, and Sit-miR156k. To characterize the chromosomal distribution of *MIR* genes in *S. italica*, we physically mapped the 159 precursor sequences across the nine chromosomes (Fig. 3). The average density was 0.42 miRNAs/Mb, with chromosomes 1 and 7 showing the highest densities (0.54 and 0.66 miRNAs/Mb, respectively). Examining their genomic distribution, most *MIR* genes were located in intergenic regions (66%), followed by intronic regions (20%), 3′ untranslated regions (3’ UTRs; 6%), coding sequences (CDSs; 4%), and 5′ untranslated regions (5’ UTRs; 3%).

### Differential expression analysis identifies water deficit–responsive miRNAs

**Fig. 4:**
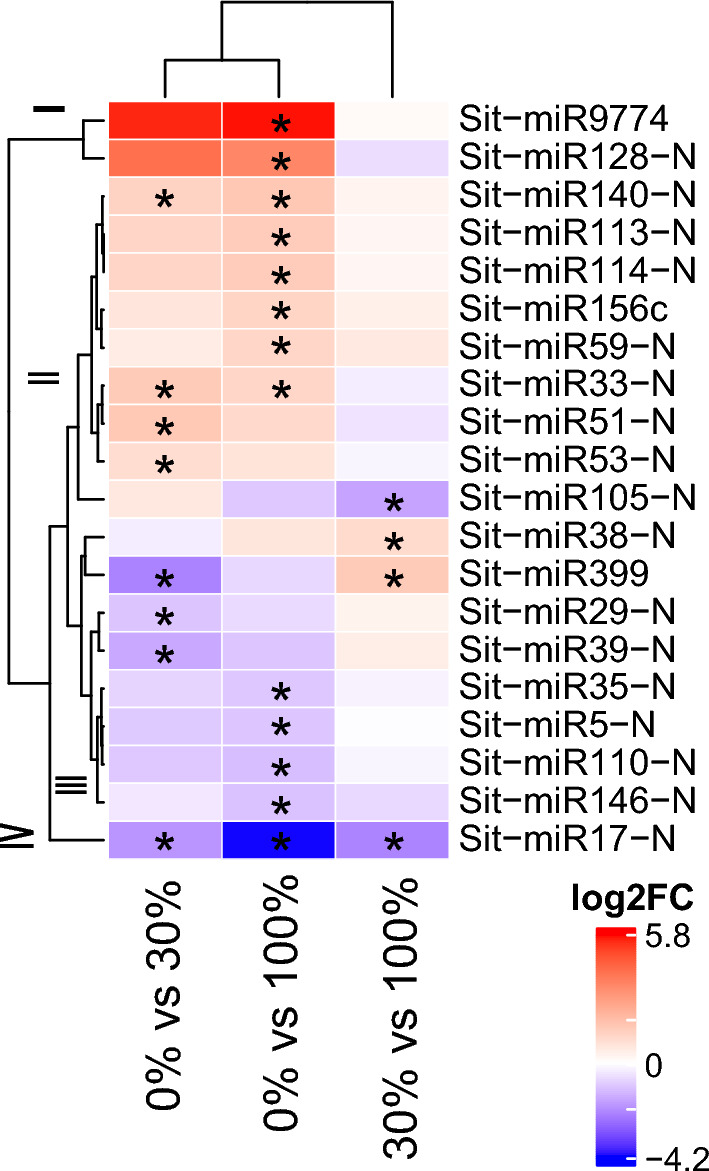
Heatmap of log_2_ fold-change (log_2_FC) values for miRNAs differentially expressed in at least one pairwise comparison among the 0%, 30%, and 100% water deficit treatments. Asterisks indicate statistically significant differentially expressed miRNAs (DEMs; |log_2_FC| ≥ 1, FDR-adjusted *P*-value ≤ 0.05). Rows (miRNAs) and columns (treatment comparisons) were hierarchically clustered using Euclidean distance

To assess the impact of water availability on miRNA regulation, we performed differential expression analysis across water-deficit treatments. A heatmap constructed from normalized CPM values revealed that sRNA-seq replicates clustered by treatment, with samples from the 30% treatment showing greater similarity to the 100% treatment than to the 0% treatment (Suppl. Fig. S2). Twenty miRNAs showed significant changes in at least one of the three pairwise comparisons (0% vs 100%, 30% vs 100%, and 0% vs 30%; Suppl. Table S6), among which three belonged to conserved miRNA families (Sit-miR399, Sit-miR9774, and Sit-miR156c), while the remaining were candidate novel miRNAs. The number of DEMs varied among the pairwise comparisons (Fig. 4). The 0% vs 100% comparison exhibited the highest number of DEMs (13), followed by 0% vs 30% (8) and 30% vs 100% (4).

The heatmap clustered the 0% vs 100% and 0% vs 30% comparisons more closely with each other than with the 30% vs 100% comparison, reinforcing that the 0% treatment induced a more divergent expression profile relative to both the 30% and 100% treatments. Hierarchical clustering of miRNAs revealed four distinct groups. The first cluster comprised two miRNAs strongly upregulated under the 0% treatment in both the 0% vs 100% and 0% vs 30% comparisons. The second cluster consisted of 8 miRNAs predominantly upregulated under severe water deficit (0% treatment). In contrast, the third cluster included miRNAs showing the opposite trend, being downregulated under the 0% treatment. The fourth cluster consisted of a single miRNA that was consistently downregulated across all comparisons. Among all DEMs, Sit-miR9774 was one of the most strongly upregulated under severe water deficit, while Sit-miR17-N was markedly downregulated across all three comparisons. miR9774 was described in *Triticum aestivum* as not responsive to water deficit (Bakhshi et al. 2017). However, more recent studies have shown that miR9774 is differentially expressed under abiotic stress, including water deficit in rice and salt stress in wheat (Singh et al. 2020; He et al. 2021), suggesting a broader and potentially conserved role in stress responses across plant species. On the other hand, the miRNA Sit-miR17-N, which is absent in miRBase and PmiREN databases, was previously predicted as nov-sit-miR152, expressed in flowers under normal growth conditions and targeting Seita.9G237800 (Yi et al. 2013), which encodes a *FERTILITY RESTORER* protein containing pentatricopeptide repeat (PPR) domain.

### Integrative in silico analysis identifies miRNA–mRNA regulatory modules associated with water-deficit response

To identify potential miRNA–mRNA regulatory modules, we initially combined in silico target prediction with integrated miRNA and target mRNA expression analysis. In silico target prediction was performed for the 159 miRNAs predicted using the psRNATarget tool (Dai et al. 2018). The target database comprised *S. italica* mRNA rtranscripts from leaves of plants subjected to the same water-deficit treatments used for the sRNA-seq analyses (Suguiyama et al. 2022). The prediction yielded 1,612 putative miRNA–mRNA modules, involving 153 miRNAs and 1094 target mRNAs, among which the majority were associated with candidate novel miRNAs (1,254), followed by conserved (335) and known (23) miRNAs (Suppl. Table S7).

**Fig. 5:**
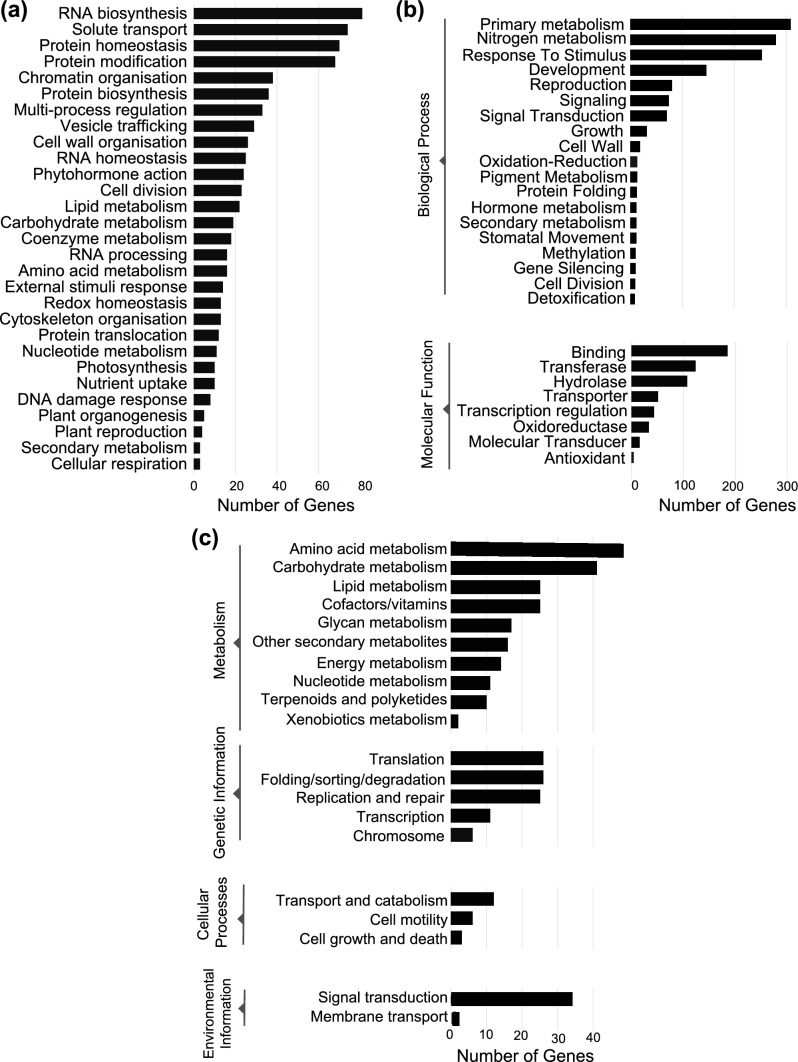
Horizontal bar plots showing the number of genes assigned to functional categories based on Mercator (**a**), Gene Ontology (GO, **b**), and KEGG (**c**) annotation. The x-axis indicates the number of genes associated with each category, while categories are displayed on the y-axis

In silico predicted targets were functionally annotated using three complementary systems: Mercator (Bolger et al. 2021), GO (Ashburner et al. 2000) and KEGG (Kanehisa and Goto 2000). Mercator-based annotation indicated that targets were assigned mainly to categories associated with distinct regulatory pathways, including gene expression control (RNA biosynthesis), proteostasis (protein modification and homeostasis), and solute transport (Fig. 5a, Suppl. Table S8). GO classified target mRNAs into categories related predominantly to basal biological processes, such as primary metabolic process, nitrogen compound metabolic process, and response to stimulus, supported by molecular functions largely associated with binding and enzymatic activities, such as transferase and hydrolase (Fig. 5b, Suppl. Table S8). KEGG-based annotation further supported these functional profiles by assigning targets to core metabolic pathways, including amino acid, carbohydrate, and lipid metabolism, as well as to pathways involved in translation, proteostasis, and DNA replication and repair (Fig. 5c, Suppl. Table S8).

To explore the potential involvement of miRNA–mRNA modules under water-deficit conditions, we analyzed the expression profiles of mRNA targets included in modules containing the 20 DEMs. We identified two miRNA–mRNA modules in which miRNA and target gene was differentially expressed in at least one treatment comparison (Table 2). The Sit-miR399/Seita.3G060600 module showed simultaneous differential expression of both miRNA and mRNA in the 30% vs 100% comparison. This target gene encodes a G-type S-receptor-like serine/threonine-protein kinase (Seita.3G060600; G-type RLK, IPR024171). Additionally, the Sit-miR39-N/Seita.8G120600 module exhibited the expected inverse expression pattern between the miRNA and its target in both the 0% vs 100% and 0% vs 30% comparisons, although the miRNA was differentially expressed only in the 0% vs 30% comparison, whereas the mRNA was differentially expressed in the 0% vs. 100% comparison. In both comparisons, the more severe water-deficit condition was associated with lower miRNA expression and higher target mRNA expression. Modules in which both components were differentially expressed within the same treatment comparison provide stronger evidence for active miRNA-mediated regulation under water deficit, whereas inverse expression patterns detected across different treatment comparisons may reflect temporal dynamics in the regulatory response, suggesting delayed target regulation or stress intensity-dependent modulation of mRNA expression.

**Table 2:** Expression profiles of miRNA and their predicted mRNA targets under different water deficit treatments

miRNA ID	miRNA log₂FC			mRNA ID	Target log₂FC		
	0% vs 100%	30% vs 100%	0% vs 30%		0% vs 100%	30% vs 100%	0% vs 30%
Sit-miR399	−0.693	1.574*	−2.267*	Seita.3G060600 (*RLK*)	−2.205*	−2.127*	−0.078
Sit-miR39-N	−1.018	0.532	−1.550*	Seita.8G120600 (*OMT)*	3.402*	0.652	2.750

*RLK* G-type RLK, *OMT* O-methyltransferase COMT-type, Asterisks indicate differentially expressed miRNA and mRNA according to the following criteria: |log₂FC|≥ 1 for both miRNAs and mRNAs

P -value ≤ 0.05 for miRNAs, and FDR-adjusted; P -value ≤ 0.05 for mRNAs

### Degradome sequencing identified miRNA–mRNA regulatory modules

Degradome sequencing was used to further identify miRNA–mRNA modules with high confidence, based on direct evidence of miRNA-guided cleavage. This analysis generated 22.1 million raw reads from a single library constructed by pooling three biological replicates from the 100%, 30%, and 0% treatment plants from the second experiment. After adapter removal and quality filtering, approximately 99.54% of the high-quality reads were retained, of which 8.6 million mapped to annotated *S. italica* transcripts. We identified 19 miRNA–mRNA modules involving 19 mRNAs and 14 miRNAs (Table 3, Suppl. Table S9), of which 11 were novel, one was known, and two were conserved. Although psRNATarget-predicted modules did not overlap with those identified by degradome sequencing, both approaches consistently found targets associated with key stress-related processes, including primary metabolism, response to stimulus, and solute transport. Functional annotation of the 19 mRNAs identified by degradome sequencing evidenced their involvement in a wide range of biological processes (Table 3). Redox-related targets were the most recurrent, including genes encoding superoxide dismutases (*CSD1/2/3*), glutathione S-transferases (*GSTF1/2/* + +), and plastidial ascorbate peroxidase *(APX7;* Jardim-Messeder et al. 2022). Targets associated with solute transport were represented by members of the ABC transporter family from both subfamilies A and G. Lipid metabolism-related targets included phospholipase D and phospho-base methyltransferase, whereas RNA-related processes comprised components involved in RNA processing and transcriptional regulation. The remaining mRNAs were annotated to protein translocation and homeostasis, protein modification, chromatin organization, cell division, plastid biogenesis, and photosynthesis categories, including a gene coding *phosphoenolpyruvate carboxylase* (*PEPC*).

**Table 3:** miRNA–mRNA modules identified in degradome sequencing through CleaveLand pipeline

miRNA	mRNA Target	Functional annotation	mRNA description
Sit-miR398_1	Seita.2G422500	Redox homeostasis	Superoxide dismutase (CSD1/2/3)
Sit-miR398_1	Seita.9G403600	Redox homeostasis	Superoxide dismutase (CSD1/2/3)
Sit-miR398_1	Seita.2G065100	Plastid biogenesis	Seedling plastid development
Sit-miRN3022a	Seita.3G062300	Cell division	MAP-kinase protein kinase (NQK/ANQ)
Sit-miR11342	Seita.9G532400	RNA processing	Sm-like protein
Sit-miR11342	Seita.7G183800	RNA biosynthesis	BBX Class IV
Sit-miR11342	Seita.9G531300	Chromatin organization	Histone (H2A/HTA)
Sit-miR3-N	Seita.5G169900	Redox homeostasis	Glutathione S-transferase (GSTF1/2/+ +)
Sit-miR22-N	Seita.5G153800	Protein translocation	SecA1 ATPase
Sit-miR32-N	Seita.5G282300	Lipid metabolism	Phospho-base methyltransferase (PEAMT1/2/3)
Sit-miR33-N	Seita.2G167500	Protein homeostasis	BiP co-chaperone Sil1
Sit-miR44-N	Seita.J017000	-	-
Sit-miR67-N	Seita.1G039200	Solute transport	ABC2 subfamily-A transporter (ABCA1/2/3)
Sit-miR74-N	Seita.1G192100	Redox homeostasis	Plastidial ascorbate peroxidase (APX)
Sit-miR85-N	Seita.7G287400	Protein modification	Alpha-1,4-fucosyltransferase
Sit-miR121-N	Seita.2G335300	Solute transport	ABC2 subfamily-G transporter (PDR1/2/3)
Sit-miR125-N	Seita.5G023200	Lipid metabolism	Phospholipase-D (PLD-zeta)
Sit-miR132-N	Seita.4G175200	Photosynthesis	PEP carboxylase (PEPC)
Sit-miR136-N	Seita.5G272600	RNA processing	RNA splicing factor (SF3A3)

**Fig. 6:**
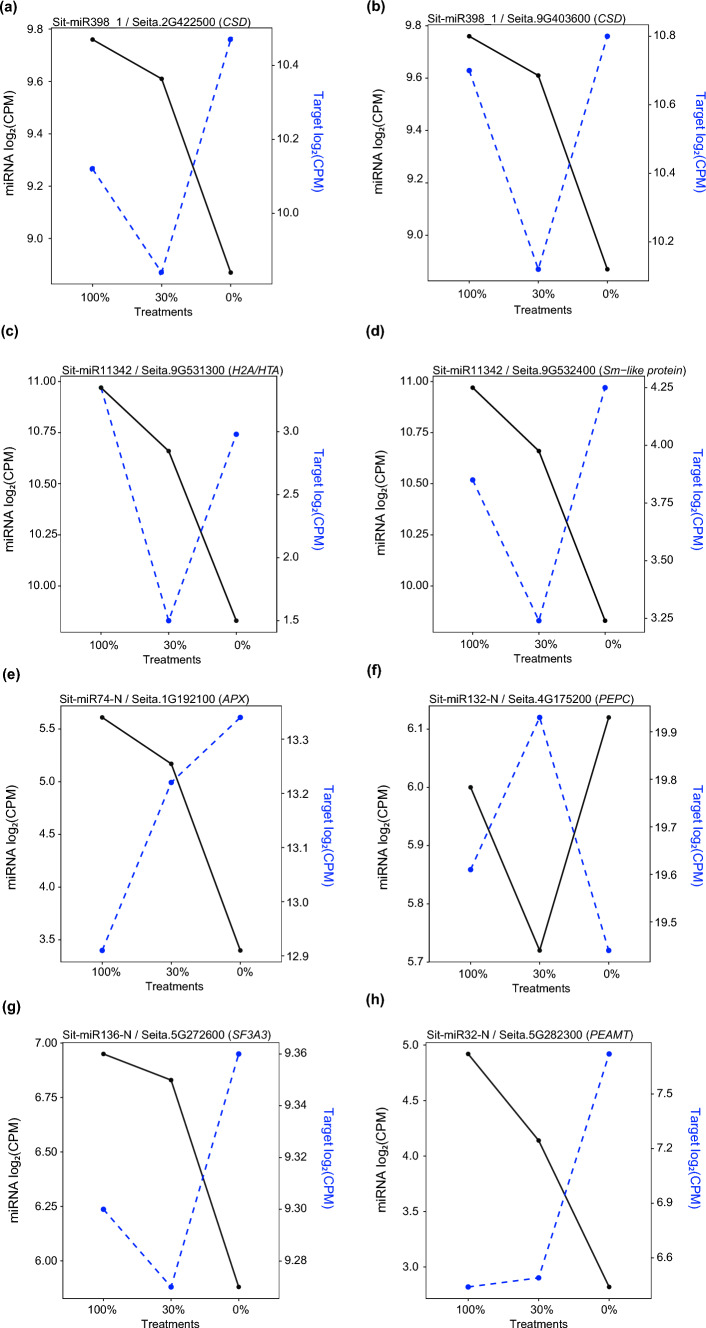
Expression profiles of miRNA-mRNA modules identified using Degradome sequencing across water deficit treatments. Each panel displays the expression patterns of miRNA-target pairs under 0%, 30%, and 100% treatments. Solid black lines represent miRNA expression levels (left y-axis, log_2_CPM), while dashed orange lines represent target gene expression levels (right y-axis, log_2_CPM). *CSD*, copper/zinc superoxide dismutase; *H2A/HTA*, histone H2A–H2B dimer; *APX7*, ascorbate peroxidase 7; *PEPC*, phosphoenolpyruvate carboxylase; *SF3A3*, RNA splicing factor 3A subcomplex component; *PEAMT*, Phosphoethanolamine N-methyltransferase

Given that miRNAs are expected to show expression patterns opposite to those of their corresponding mRNA targets, we next examined the expression profiles of the miRNA–mRNA modules identified by degradome sequencing. Among the 19 modules, eight exhibited the expected antagonistic pattern between miRNAs and their targets (Fig. 6). These mRNAs code for proteins involved in oxidative stress response (the *superoxide dismutases CSD1/2/3* and *APX7*), chromatin organization (*histone H2A/HTA*), RNA metabolism (the *Sm-like protein* and the *RNA splicing factor SF3A3*), carbon metabolism (the *phosphoenolpyruvate carboxylase* PEPC), and phospholipid biosynthesis (the *phosphobase methyltransferases PMT1/2/3*). Under 0% treatment, miRNAs and their targets showed a comparatively greater divergence in expression levels, suggesting that miRNA-mediated regulation may become more pronounced under the severe treatment conditions applied here. The conserved miRNAs Sit-miR398_1 (Fig. 6a-b) and Sit-miR1342 (Fig. 6c-d) were found to regulate a pair of target mRNAs, all of which displayed similar expression trends across treatments, reinforcing the consistency of their regulatory profiles. Antagonistic expression patterns were particularly evident among modules involving candidate novel miRNAs (Fig. 6e-h). Together, the prediction, expression profiling, and degradome validation analyses reveal a complex regulatory landscape in which water deficit–responsive miRNAs potentially modulate mRNAs involved in redox balance, metabolism, and cellular homeostasis.

### Transient expression assays support the interaction between novel miRNAs and water deficit–related targets

**Fig. 7:**
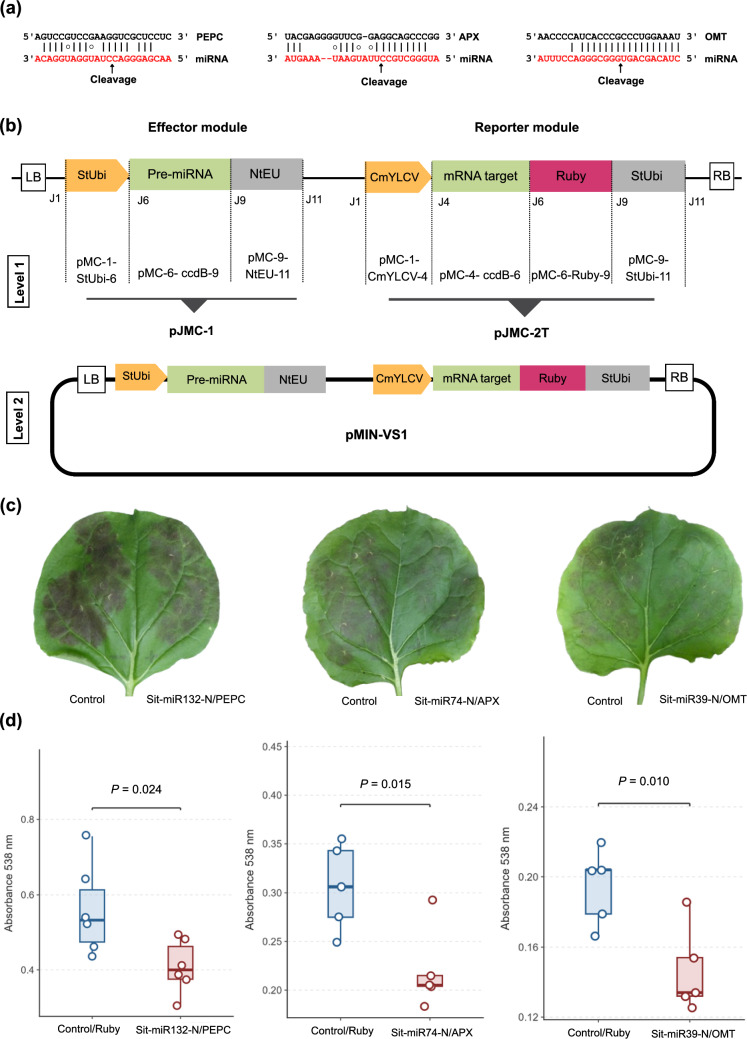
Interaction assays of miRNA–mRNA modules using a Ruby-based reporter system. (**a**) Sequence alignment between each candidate miRNA and its corresponding target site. Sequence complementarity, G–U wobble interactions, and predicted cleavage sites are indicated by vertical bars, circles, and vertical arrows between nucleotides 10 and 11 from the miRNA 5′ end, respectively. (**b**) Schematic representation of the constructs. The experimental and control constructs share the same basic structure. In the experimental constructs, the effector module contains the pre-miRNA sequence, whereas in the control constructs, it contains the GFP coding sequence. (**c**) Representative images of infiltrated *N. benthamiana* leaves showing betalain accumulation (purple spots). Co-expression of each miRNA with its respective target resulted in reduced pigmentation compared to the control, indicating potential interactions. (**d**) Betalain levels measured after extraction from leaf discs from infiltrated areas. For each construct, three biological and two technical replicates were analyzed (Student’s *t*-test; *P* ≤ 0.05)

To gain further evidence that the identified miRNAs could inhibit their target mRNAs, we selected three modules for miRNA–mRNA interaction assays in agroinfiltrated *N. benthamiana* leaves. Of the three modules, two were validated by degradome sequencing and involved novel miRNAs and their respective mRNA targets: Sit-miR74-N/Seita.1G192100 (*APX7*) and Sit-miR132-N/Seita.4G175200 (*PEPC*). The third, Sit-miR39-N/Seita.8G120600 (*OMT*), was predicted by psRNATarget and likewise involved a candidate novel miRNA. Additionally, these targets were selected based on their functional relevance to key water deficit–associated processes, including photosynthesis (*PEPC*), oxidative stress response (*APX7*), and flavonoid-related pathways (*OMT*). The design of the cassettes used in this assay was based on the predicted sequence alignment between each candidate miRNA and its respective target site, identified during the target prediction analysis (Fig. 7a). This alignment revealed the putative miRNA binding region and predicted cleavage site, which guided the construction of the reporter vectors (Fig. 7b). The expression cassette comprised an effector module, in which the candidate miRNA was overexpressed using the *Solanum tuberosum UBIQUITIN* promoter, and a reporter module, in which the RUBY reporter fused in frame with the cleavage site of the target mRNA was overexpressed by the Cestrum yellow leaf curling virus (CmYLCV) promoter. GFP was used as negative control in the effector module. For all three modules, co-expression of the miRNA precursor with its respective target sequence resulted in visibly reduced betalain accumulation in infiltrated *N. benthamiana* leaves compared to the corresponding negative controls, indicating repression of the RUBY reporter driven by the target cleavage sequence (Fig. 7c). Quantification of betalain levels confirmed a significant reduction in reporter signal for all tested miRNA–target modules (Student’s t-test, *P* ≤ 0.05; Fig. 7d), providing functional evidence that these miRNAs effectively recognize and repress their predicted targets. Together, these results provide experimental validation for candidate novel miRNA–mRNA modules and support their role as active post-transcriptional regulators in the water-deficit response of *S. italica*.

## Discussion

Drought is a major constraint on crop productivity, triggering physiological and morphological adjustments that affect plant growth and reproduction (Lata et al. 2015). In the context of food production and biomass generation for biofuels, drought can cause economic losses and compromise food security (Yadav et al. 2020). The magnitude of these impacts is reflected in the significant yield reductions reported for major crops under drought, including wheat (up to 64.5%), maize (63–87%), rice (53–92%), and soybean (46–71%) (Hussain et al. 2019). Water-deficit responses are mediated by a complex molecular network that coordinates the tradeoff between survival and growth through transcriptional and post-transcriptional regulatory layers (Lata et al. 2015; Kumar et al. 2025). Within this network, miRNAs act as key regulators by fine-tuning transcript levels and, consequently, protein accumulation (Rathod and Puri 2026). To explore this regulatory landscape, our study comprised the analysis of two independent experiments performed under the same experimental design, thereby ensuring consistency across analyses. The first generated the sRNA-seq and RNA-seq datasets used to identify water deficit–responsive miRNAs and their predicted targets, whereas the second produced degradome libraries for the validation of miRNA–mRNA interactions.

Building on this integrative framework, the analysis of sRNA-seq libraries enabled the identification of 159 mature miRNAs in *S. italica* leaves, with the majority corresponding to newly predicted miRNAs. The identification of miRNAs from 11 conserved families confirms the recovery of previously reported miRNAs, while the large number of newly predicted miRNAs expands the current repertoire of sRNAs of *S. italica*. The high number of novel miRNAs may be explained by the absence of deposited miRNA records for the genus *Setaria* in miRBase, the principal public repository for miRNA annotation. Complementary information, including homology relationships (Fig. 2) and a predominant distribution of *MIR* genes in intergenic regions, revealed patterns consistent with those previously reported for Poaceae species (Merchan et al. 2009; Yi et al. 2013; Yue et al. 2021).

Long-term water deficit imposed constraints on both vegetative growth and reproductive development in *S. italica*, with severe stress (0% treatment) preventing the transition to the reproductive stage and moderate stress (30% treatment) causing milder developmental impacts (Fig. 1; Table 1). Although both water-deficit experiments showed consistent reductions in vegetative growth and reproductive performance, significant treatment versus experiment interactions were detected for shoot biomass, RWC, and panicle weight (Suppl. Table S2). These interactions may reflect subtle biological responses and technical differences between experiments, including variation in growth chamber conditions and in the equipment used to monitor soil moisture, which may also have contributed to differences in sampling timing. Nevertheless, both experiments showed significant and comparable effects of water deficit on vegetative and reproductive traits. Accordingly, the observed experimental variation is unlikely to have resulted in the identification of false-positive miRNA–mRNA modules, particularly because degradome was obtained from an independent experiment, reducing the likelihood that these modules reflect experiment-specific effects.

The responses of *S. italica* to water deficit provided an experimental framework for investigating the underlying molecular regulatory mechanisms, with emphasis on miRNAs. Among the miRNA–mRNA modules identified through in silico target prediction and differential expression analyses, one exhibited a consistent negative correlation between miRNA and target mRNA expression levels. The module Sit-miR399/Seita.3G060600 showed a significant inverse expression pattern in the 30% vs 100% comparison, with Sit-miR39 upregulated and Seita.3G060600 downregulated (Table 2). The involvement of miRNAs of the miR399 family in abiotic stress responses has been previously reported in different plant species (Liu et al. 2019). For instance, members of miR399 family were upregulated in maize and wheat under water deficit (Akdogan et al. 2016; Liu et al. 2019). miR399 targets are commonly associated with phosphorus metabolism and signaling, including enzymes such as inorganic pyrophosphatases (Ferreira et al. 2012). Consistent with this observation, the predicted target Seita.3G060600 encodes a protein belonging to the G-type RLK family, whose members are involved in the perception of abiotic and biotic stress signals and in the initiation of intracellular signaling cascades (Sun et al. 2020). Thus, this locus emerges as a promising candidate for future studies on water-deficit responses.

We also identified a module in which both miRNAs and their targets were differentially expressed in water-deficit treatments, although not within the same comparisons. In the module Sit-miR39-N/Seita.8G120600 (*OMT*), the miRNA was downregulated in the 0% vs 30% comparison, whereas the Seita.8G120600 locus was upregulated in the 0% vs 100% comparison (Table 2), suggesting that this module responded more pronouncedly to severe water deficit. The interaction between Sit-miR39-N and Seita.8G120600 was validated by transient expression assay (Fig. 7d), while the inverse expression pattern observed between the miRNA and its target mRNA across water-deficit treatments further supports context-dependent regulation. Seita.8G120600 codes for an *O-methyltransferase COMT-type* (*OMT*), involved in flavonoid metabolism as evidenced by the GO functional annotation (Suppl. Table S8). Flavonoids play a central role in water-deficit response by protecting the photosynthetic machinery (Ahmed et al. 2021). Transcriptome analyses from the same experimental design identified secondary metabolism, particularly flavonoid-related pathways, as one of the most responsive categories under water deficit in *S. italica* (Suguiyama et al. 2022).

Degradome sequencing was employed to provide experimental validation of miRNA-guided cleavage events and to assess their contribution to the transcriptional landscape under water deficit in *S. italica*. Several modules showing trends of inverse expression patterns between miRNAs and their target mRNAs were validated by degradome sequencing (Fig. 6; Table 3), lending further support to their involvement in physiological processes related to water-deficit response. Because the RNA-seq and sRNA-seq samples were collected 38 days after treatment initiation, whereas the degradome samples were collected at 51 days, we cannot exclude the possibility that the lack of significant differential expression in degradome-supported miRNA–mRNA modules may, at least in part, reflect differences in sampling time. Nevertheless, soil moisture conditions and plant developmental stages were similar in both experiments, indicating that the plants were exposed to comparable physiological and developmental contexts.

The identified mRNA targets validated by the degradome sequencing were associated with diverse biological processes, spanning transcriptional, metabolic, and physiological adjustments (including photosynthesis and redox homeostasis), suggesting that miRNA-mediated regulation may contribute to multiple layers of the water-deficit response. Among these modules, miR398/CSD1/2/3 is particularly notable because of its functional relevance. It involves the highly conserved miRNA family miR398, widely recognized as a master regulator of oxidative stress responses, with multiple experimentally validated targets including the *copper/zinc superoxide dismutases CSD1/2/3* (Li et al. 2022). The miR398/CSD1/2/3 module is highly conserved across angiosperms and plays a well-established role in abiotic stresses responses, as demonstrated in *Arabidopsis*, rice, wheat, tomato, maize, and sorghum, among other species (Li et al. 2022). Under stress conditions, this module prevents the excessive accumulation of superoxide (O₂⁻) by regulating CSD1/2/3 expression via miR398 activity (Li et al. 2022). In *S. italica*, we identified two Sit-miR398_1/CSD1/2/3 modules, each containing a different CSD1/2/3 locus, both displaying a similar expression profile in response to water deficit (Fig. 6a-b). Under the 0% treatment, miR398 was downregulated, accompanied by upregulation of *CSD1/2/3* loci, likely reflecting a regulatory response that contributes to water-deficit tolerance. Consistently, drought-tolerant maize lines also exhibited miR398 downregulation under severe water deficit (Liu et al. 2019; Seeve et al. 2019), further supporting a role for this module in water-deficit tolerance in *S. italica*.

Two modules identified with degradome sequencing were further validated using transient expression assays, Sit-miR74-N/*APX7* and Sit-miR132-N/*PEPC* (Fig. 7). *APX7* belongs to the ascorbate peroxidase gene family widely reported to be regulated at both transcriptional and post-transcriptional levels in response to water deficit, playing a key role in ROS scavenging (Yoshimura and Ishikawa 2024). The APX7 identified in our study is predicted to localize chloroplasts/mitochondria (Jardim-Messeder et al. 2022), suggesting a potential role in protecting photosynthetic and respiratory metabolism during water deficit. In agreement with this hypothesis, Sit-miR74-N expression progressively decreased with increasing water-deficit severity, whereas *APX7* expression showed a positive association with water-deficit intensity (Fig. 6e), supporting a regulatory interaction linked to oxidative stress management under dehydration conditions. The relevance of chloroplast-associated APXs during water deficit has also been demonstrated in rice. Silencing of the thylakoidal *APX8* resulted in increased H_2_O_2_ accumulation, membrane damage, disruption of redox homeostasis, and stronger photosynthetic limitations under mild water deficit (Cunha et al. 2019). These effects included reductions in stomatal conductance, CO_2_ assimilation, and electron transport capacity, highlighting the importance of chloroplast APXs not only in ROS scavenging but also in maintaining photosynthetic performance and redox signaling during water deficit.

In the second module, Sit-miR132-N was suggested to regulate the *phosphoenolpyruvate carboxylase* (*PEPC*) gene, which encodes an important enzyme in C4 photosynthesis related to carbon fixation, energy balance, and nitrogen assimilation (Liu et al. 2017). Previous studies using transgenic rice and wheat expressing the maize *PEPC* gene reported improved water-deficit tolerance (Qin et al. 2016; Liu et al. 2017). In *S. italica*, Sit-miR132-N was downregulated and *PEPC* upregulated under moderate stress (30% treatment), whereas the opposite pattern was observed under severe stress (0% treatment) (Fig. 6f). This response agrees with previous findings in *S. italica* showing that *PEPC* was associated with the maintenance of photosynthesis and stomatal conductance under moderate water deficit (Suguiyama et al. 2022). Under severe stress, however, the inverse expression pattern suggests repression of *PEPC*, likely reflecting a state of substrate and energy limitation, possibly preceding stress induced senescence.

The expectation that miRNAs and their corresponding mRNA targets should display inverse expression patterns may represent a useful criterion for filtering and prioritizing candidate loci in genome-wide datasets derived from high-throughput sequencing analyses. Nevertheless, functional miRNA–mRNA modules may also exist even when such inverse expression patterns are not detected. For example, mRNAs regulated through miRNA-mediated translational repression, rather than target cleavage, may not exhibit reduced read abundance in RNA-seq datasets. Moreover, expression patterns associated with cell-type-specific regulatory modules may be obscured in bulk RNA-seq analyses. Although we focused here on negative expression associations because of the large dataset generated, several additional modules remain to be further explored using complementary methodologies, such as single-cell RNA-seq. Such approaches may provide valuable insights into the regulatory mechanisms underlying gene expression responses to water deficit in C4 photosynthetic Poaceae.

In conclusion, this study expands our current knowledge of miRNA-mediated post-transcriptional regulation in *S. italica* by identifying previously undescribed miRNAs expressed under long-term water deficit. We also identified potential mRNA targets of these miRNAs, highlighting the likely contribution of these regulatory modules to water-deficit responses. Our results indicate that miRNA-mediated regulation under water deficit involves both well-established stress-responsive processes, such as redox homeostasis and photosynthetic regulation, and less explored pathways, including chromatin organization and cell division. It is important to note that future functional studies should further evaluate the differential expression trends observed for the miRNA–mRNA modules under water-deficit conditions, especially for Sit-miR132-N/*PEPC* and Sit-miR74-N/*APX7*. Together, our findings indicate that miRNA-mediated regulation contributes to multiple aspects of the water-deficit response, encompassing processes related to signaling, transcriptional control, metabolism, and physiological adjustment.

## Supplementary Information

Below is the link to the electronic supplementary material.

Supplementary file1 (XLSX 980 KB)

Supplementary file2 (XLSX 19 KB)

Supplementary file3 (PDF 252 KB)

Supplementary file4 (PDF 264 KB)

Supplementary file5 (XLSX 26 KB)

Supplementary file6 (DOCX 10 KB)

Supplementary file7 (DOCX 10 KB)

Supplementary file8 (DOCX 10 KB)

Supplementary file9 (XLSX 37 KB)

Supplementary file10 (DOCX 784 KB)

Supplementary file11 (XLSX 118 KB)

## Data Availability

The data that support the findings of this study are openly available in NCBI Bioproject (https://www.ncbi.nlm.nih.gov/), reference numbers PRJNA593563 and PRJNA1450095.

## Abbreviations

APX7: Plastidial ascorbate peroxidase
DEMs: Differentially expressed miRNAs
RWC: Relative water content
