# Supplementary material for: Integrative RNA sequencing uncovers the miRNA–mRNA landscape of water-deficit response in Setaria italica
Source: Planta. 2026 Aug 10;264(3):92. doi: 10.1007/s00425-026-05117-5 (PMC13457271; doi:10.1007/s00425-026-05117-5)
Supplement: Supplementary file 1 [file 425_2026_5117_MOESM3_ESM.pdf]

•**Supplementary Information**
•**Article title:** Integrative RNA sequencing uncovers a multilayer miRNA–mRNA modules landscape of water deficit response in *Setaria italica*
•**Journal:** Planta
•**Authors:** M.R. Juliana[1], G.B.T. Vitor[1], G. Gabriel[2], F.O. Leandro[2], C. Igor[2], S. Nathalia[1]
•**Affiliations:** [1]Centro de Ciências Naturais e Humanas (CCNH), Universidade Federal do ABC (UFABC), São Bernardo do Campo, SP, Brazil. [2]LigninLab, Department of Botany, Institute of Biosciences, University of São Paulo, São Paulo, SP, Brazil.
•**Corresponding author:** Nathalia de Setta; Email: nathalia.setta@ufabc.edu.br.

Suppl. Fig. S1

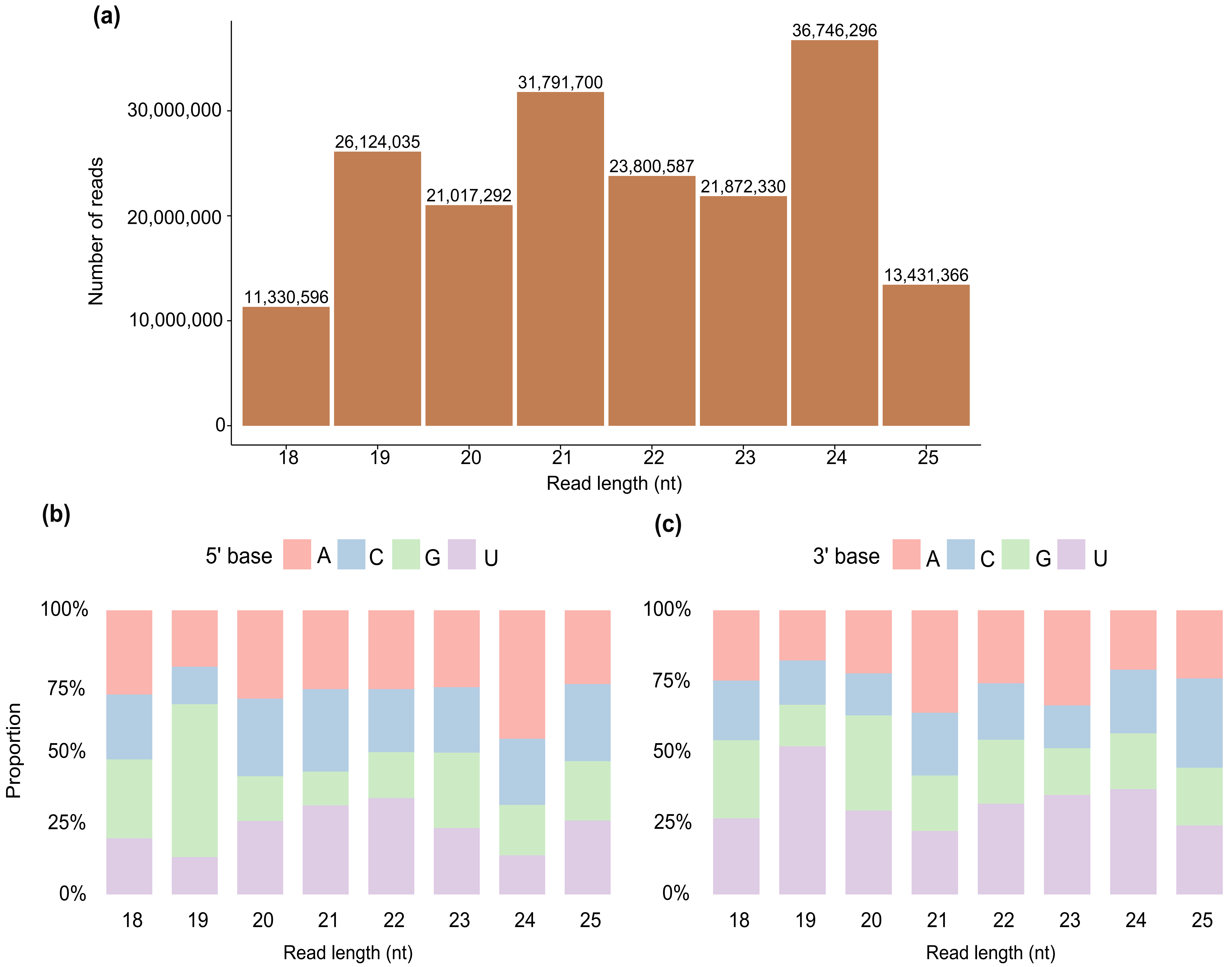

Fig. S1 Size distribution and nucleotide composition of sRNAs from *S. italica.* (**a**) Length distribution of filtered reads. (**b**) 5′ terminal nucleotide composition of non-rRNA and non-tRNA cleaned sRNA reads. (**c**) 3′ terminal nucleotide composition of non-rRNA and non-tRNA cleaned sRNA reads
