# Supplementary material for: Integrative RNA sequencing uncovers the miRNA–mRNA landscape of water-deficit response in Setaria italica
Source: Planta. 2026 Aug 10;264(3):92. doi: 10.1007/s00425-026-05117-5 (PMC13457271; doi:10.1007/s00425-026-05117-5)
Supplement: Supplementary file 3 [file 425_2026_5117_MOESM10_ESM.docx]

**Supplementary information for:** Integrative RNA sequencing uncovers a multilayer miRNA–mRNA modules landscape of water deficit response in *Setaria italica*

**Journal:** Planta

**Authors:** M.R. Juliana¹, G.B.T. Vitor¹, G. Gabriel², F.O. Leandro², C. Igor², S. Nathalia¹

**Affiliations:** ¹Centro de Ciências Naturais e Humanas (CCNH), Universidade Federal do ABC (UFABC), São Bernardo do Campo, SP, Brazil. ²LigninLab, Department of Botany, Institute of Biosciences, University of São Paulo, São Paulo, SP, Brazil.

**Corresponding author:** Nathalia de Setta; Email: nathalia.setta@ufabc.edu.br.

**Table S6** miRNAs expressed in *S. italica* submitted to the water deficit experiment. Values in bold indicate differentially expressed miRNAs (|log₂FC| ≥ 1 and FDR-adjusted *P*-value ≤ 0.05)

| miRNA | 0% x 100% | | 30% x 100% | | | 0% x 30% | | |
| --- | --- | --- | --- | --- | --- | --- | --- | --- |
|  | LogFC | *P*-adj | | LogFC | *P*-adj | | LogFC | *P*-adj |
| Sit-miR11342 | -0.094 | 0.703 | | 0.041 | 0.867 | | -0.135 | 0.586 |
| Sit-miR156j | -0.419 | 0.175 | | -0.116 | 0.684 | | -0.303 | 0.309 |
| Sit-miR156c | **1.273** | **0.021** | | 0.469 | 0.296 | | 0.804 | 0.097 |
| Sit-miR156k | -0.378 | 0.358 | | 0.084 | 0.831 | | -0.462 | 0.271 |
| Sit-miR166 | 0.028 | 0.971 | | 0.547 | 0.497 | | -0.519 | 0.518 |
| Sit-miR167 | 0.469 | 0.313 | | -0.190 | 0.670 | | 0.659 | 0.173 |
| Sit-miR171_1 | 0.388 | 0.282 | | -0.517 | 0.167 | | 0.905 | 0.034 |
| Sit-miR171f | 0.517 | 0.116 | | 0.336 | 0.279 | | 0.182 | 0.544 |
| Sit-miR1848 | -0.098 | 0.785 | | 0.124 | 0.721 | | -0.223 | 0.544 |
| Sit-miR395k | 0.797 | 0.191 | | 0.952 | 0.119 | | -0.155 | 0.780 |
| Sit-miR395_2 | -0.006 | 0.987 | | 0.257 | 0.509 | | -0.264 | 0.503 |
| Sit-miR395_1 | -0.006 | 0.987 | | 0.257 | 0.509 | | -0.264 | 0.503 |
| Sit-miR397 | -0.660 | 0.127 | | -0.172 | 0.661 | | -0.488 | 0.238 |
| Sit-miR398_3 | 0.761 | 0.321 | | 0.819 | 0.289 | | -0.057 | 0.937 |
| Sit-miR398_2 | 1.485 | 0.050 | | 0.194 | 0.758 | | 1.291 | 0.078 |
| Sit-miR398_1 | 0.223 | 0.806 | | 0.612 | 0.508 | | -0.390 | 0.670 |
| Sit-miR399 | -0.693 | 0.128 | | **1.574** | **0.002** | | **-2.267** | **0.001** |
| Sit-miR535 | 0.972 | 0.266 | | -0.535 | 0.523 | | 1.508 | 0.109 |
| Sit-miR9774 | **5.722** | **0.027** | | 0.177 | 0.917 | | 5.545 | 0.029 |
| Sit-miRN3003 | -0.016 | 0.951 | | -0.349 | 0.211 | | 0.333 | 0.231 |
| Sit-miRN3022a | -0.080 | 0.818 | | -0.695 | 0.080 | | 0.615 | 0.124 |
| Sit-miRN3022b | -0.080 | 0.818 | | -0.695 | 0.080 | | 0.615 | 0.124 |
| Sit-miRN3029a | -0.002 | 0.994 | | -0.405 | 0.190 | | 0.403 | 0.193 |
| Sit-miRN3029b | -0.002 | 0.994 | | -0.405 | 0.190 | | 0.403 | 0.193 |
| Sit-miR1-N | -0.069 | 0.845 | | -0.258 | 0.476 | | 0.188 | 0.599 |
| Sit-miR10-N | -0.126 | 0.656 | | -0.244 | 0.395 | | 0.118 | 0.677 |
| Sit-miR100-N | 0.029 | 0.934 | | 0.250 | 0.479 | | -0.221 | 0.543 |
| Sit-miR101-N | -0.633 | 0.089 | | -0.107 | 0.740 | | -0.526 | 0.145 |
| Sit-miR102-N | 0.098 | 0.798 | | -0.054 | 0.883 | | 0.152 | 0.696 |
| Sit-miR103-N | -0.403 | 0.121 | | -0.105 | 0.652 | | -0.297 | 0.232 |
| Sit-miR104-N | -0.711 | 0.041 | | -0.159 | 0.581 | | -0.552 | 0.092 |
| Sit-miR105-N | -0.981 | 0.150 | | **-1.667** | **0.032** | | 0.686 | 0.340 |
| Sit-miR106-N | -0.348 | 0.285 | | -0.278 | 0.373 | | -0.070 | 0.823 |
| Sit-miR107-N | 0.228 | 0.596 | | 0.342 | 0.422 | | -0.114 | 0.789 |
| Sit-miR108-N | 0.188 | 0.709 | | -0.428 | 0.410 | | 0.616 | 0.258 |
| Sit-miR109-N | 0.603 | 0.153 | | -0.144 | 0.707 | | 0.747 | 0.092 |
| Sit-miR11-N | -0.892 | 0.039 | | -0.215 | 0.539 | | -0.677 | 0.094 |
| Sit-miR110-N | **-1.175** | **0.024** | | -0.182 | 0.631 | | -0.993 | 0.046 |
| Sit-miR111-N | -0.109 | 0.841 | | 0.763 | 0.171 | | -0.872 | 0.142 |
| Sit-miR112-N | 1.545 | 0.314 | | 1.323 | 0.378 | | 0.222 | 0.872 |
| Sit-miR113-N | **1.536** | **0.041** | | 0.274 | 0.657 | | 1.262 | 0.077 |
| Sit-miR114-N | **1.536** | **0.041** | | 0.274 | 0.657 | | 1.262 | 0.077 |
| Sit-miR115-N | -0.597 | 0.170 | | 0.037 | 0.927 | | -0.634 | 0.149 |
| Sit-miR116-N | 0.433 | 0.257 | | -0.503 | 0.197 | | 0.936 | 0.040 |
| Sit-miR117-N | -0.132 | 0.717 | | 0.035 | 0.920 | | -0.168 | 0.648 |
| Sit-miR118-N | 0.072 | 0.872 | | -0.301 | 0.495 | | 0.373 | 0.427 |
| Sit-miR120-N | 0.072 | 0.872 | | -0.301 | 0.495 | | 0.373 | 0.427 |
| Sit-miR121-N | -0.149 | 0.783 | | -0.058 | 0.911 | | -0.092 | 0.867 |
| Sit-miR122-N | -0.152 | 0.740 | | 0.249 | 0.556 | | -0.401 | 0.395 |
| Sit-miR123-N | 0.309 | 0.242 | | 0.032 | 0.896 | | 0.277 | 0.291 |
| Sit-miR124-N | 0.153 | 0.672 | | 0.091 | 0.793 | | 0.061 | 0.865 |
| Sit-miR125-N | 0.057 | 0.857 | | 0.430 | 0.196 | | -0.373 | 0.262 |
| Sit-miR126-N | -0.117 | 0.722 | | -0.039 | 0.904 | | -0.078 | 0.812 |
| Sit-miR127-N | 0.134 | 0.600 | | -0.129 | 0.611 | | 0.262 | 0.321 |
| Sit-miR128-N | **3.535** | **0.037** | | -0.598 | 0.648 | | 4.134 | 0.023 |
| Sit-miR129-N | 0.728 | 0.192 | | 0.197 | 0.697 | | 0.531 | 0.328 |
| Sit-miR13-N | 0.319 | 0.268 | | 0.093 | 0.731 | | 0.226 | 0.422 |
| Sit-miR130-N | -0.349 | 0.219 | | -0.144 | 0.585 | | -0.205 | 0.453 |
| Sit-miR131-N | 0.248 | 0.676 | | -1.198 | 0.098 | | 1.446 | 0.061 |
| Sit-miR132-N | 1.335 | 0.050 | | 0.118 | 0.834 | | 1.217 | 0.068 |
| Sit-miR133-N | 0.369 | 0.301 | | 0.044 | 0.895 | | 0.325 | 0.359 |
| Sit-miR134-N | -1.248 | 0.122 | | -0.890 | 0.203 | | -0.358 | 0.649 |
| Sit-miR135-N | 0.227 | 0.763 | | 0.065 | 0.929 | | 0.161 | 0.830 |
| Sit-miR136-N | -0.021 | 0.939 | | 0.231 | 0.396 | | -0.251 | 0.365 |
| Sit-miR137-N | -1.537 | 0.078 | | -0.819 | 0.294 | | -0.718 | 0.354 |
| Sit-miR138-N | 0.717 | 0.049 | | 0.511 | 0.126 | | 0.206 | 0.508 |
| Sit-miR139-N | 0.794 | 0.124 | | 0.642 | 0.192 | | 0.151 | 0.743 |
| Sit-miR14-N | -0.334 | 0.453 | | -0.167 | 0.697 | | -0.167 | 0.704 |
| Sit-miR140-N | **1.670** | **0.007** | | 0.334 | 0.453 | | **1.336** | **0.018** |
| Sit-miR142-N | -0.142 | 0.672 | | -0.078 | 0.807 | | -0.063 | 0.850 |
| Sit-miR143-N | 0.018 | 0.948 | | 0.082 | 0.765 | | -0.064 | 0.814 |
| Sit-miR144-N | 0.143 | 0.566 | | 0.070 | 0.775 | | 0.073 | 0.768 |
| Sit-miR145-N | 0.376 | 0.264 | | 0.143 | 0.655 | | 0.232 | 0.475 |
| Sit-miR146-N | **-1.110** | **0.023** | | -0.691 | 0.107 | | -0.419 | 0.295 |
| Sit-miR147-N | 0.125 | 0.757 | | -0.381 | 0.351 | | 0.506 | 0.241 |
| Sit-miR15-N | 0.056 | 0.910 | | 0.742 | 0.166 | | -0.685 | 0.201 |
| Sit-miR16-N | -0.191 | 0.483 | | -0.253 | 0.355 | | 0.061 | 0.819 |
| Sit-miR17-N | **-4.168** | **0.001** | | **-2.234** | **0.010** | | **-1.934** | **0.034** |
| Sit-miR18-N | -0.135 | 0.637 | | -0.073 | 0.794 | | -0.062 | 0.827 |
| Sit-miR19-N | -0.135 | 0.641 | | -0.051 | 0.856 | | -0.084 | 0.773 |
| Sit-miR2-N | 0.364 | 0.275 | | 0.136 | 0.669 | | 0.228 | 0.481 |
| Sit-miR20-N | 0.079 | 0.779 | | 0.152 | 0.585 | | -0.073 | 0.795 |
| Sit-miR21-N | 0.079 | 0.779 | | 0.152 | 0.585 | | -0.073 | 0.795 |
| Sit-miR22-N | -0.668 | 0.102 | | 0.005 | 0.990 | | -0.673 | 0.102 |
| Sit-miR23-N | 0.351 | 0.352 | | 0.476 | 0.207 | | -0.125 | 0.731 |
| Sit-miR24-N | 0.381 | 0.368 | | 0.234 | 0.559 | | 0.147 | 0.722 |
| Sit-miR25-N | 0.078 | 0.877 | | -0.615 | 0.238 | | 0.694 | 0.213 |
| Sit-miR26-N | 0.810 | 0.091 | | 0.490 | 0.263 | | 0.320 | 0.456 |
| Sit-miR27-N | -0.147 | 0.757 | | -0.134 | 0.769 | | -0.013 | 0.977 |
| Sit-miR28-N | -0.620 | 0.207 | | -0.586 | 0.209 | | -0.034 | 0.945 |
| Sit-miR29-N | -0.659 | 0.097 | | 0.380 | 0.264 | | **-1.039** | **0.021** |
| Sit-miR3-N | 0.071 | 0.755 | | -0.016 | 0.944 | | 0.087 | 0.703 |
| Sit-miR30-N | 0.520 | 0.334 | | 0.346 | 0.490 | | 0.174 | 0.738 |
| Sit-miR31-N | 0.259 | 0.598 | | 0.168 | 0.726 | | 0.092 | 0.851 |
| Sit-miR32-N | -0.657 | 0.208 | | -0.417 | 0.388 | | -0.240 | 0.633 |
| Sit-miR33-N | **1.235** | **0.046** | | -0.341 | 0.529 | | **1.577** | **0.021** |
| Sit-miR34-N | 0.399 | 0.521 | | 0.957 | 0.137 | | -0.559 | 0.369 |
| Sit-miR35-N | **-1.004** | **0.038** | | -0.231 | 0.532 | | -0.773 | 0.093 |
| Sit-miR36-N | 0.134 | 0.850 | | 0.149 | 0.817 | | -0.015 | 0.981 |
| Sit-miR37-N | 0.719 | 0.233 | | 1.217 | 0.052 | | -0.498 | 0.375 |
| Sit-miR38-N | 0.753 | 0.106 | | **1.095** | **0.025** | | -0.342 | 0.405 |
| Sit-miR39-N | -1.018 | 0.093 | | 0.532 | 0.283 | | **-1.550** | **0.022** |
| Sit-miR4-N | -0.334 | 0.234 | | -0.250 | 0.359 | | -0.084 | 0.752 |
| Sit-miR41-N | 0.177 | 0.592 | | 0.493 | 0.154 | | -0.316 | 0.349 |
| Sit-miR42-N | -0.004 | 0.995 | | -0.114 | 0.826 | | 0.110 | 0.836 |
| Sit-miR43-N | 0.852 | 0.186 | | -0.214 | 0.719 | | 1.066 | 0.115 |
| Sit-miR44-N | -0.247 | 0.450 | | 0.397 | 0.219 | | -0.643 | 0.079 |
| Sit-miR45-N | 0.678 | 0.080 | | 0.547 | 0.133 | | 0.131 | 0.698 |
| Sit-miR46-N | -0.346 | 0.356 | | -0.498 | 0.188 | | 0.152 | 0.680 |
| Sit-miR47-N | 0.128 | 0.817 | | 0.098 | 0.855 | | 0.030 | 0.957 |
| Sit-miR48-N | -0.626 | 0.158 | | 0.186 | 0.620 | | -0.812 | 0.082 |
| Sit-miR5-N | **-1.020** | **0.025** | | -0.062 | 0.862 | | -0.958 | 0.032 |
| Sit-miR51-N | 1.151 | 0.105 | | -0.493 | 0.462 | | **1.644** | **0.042** |
| Sit-miR52-N | 0.259 | 0.474 | | 0.483 | 0.195 | | -0.224 | 0.533 |
| Sit-miR53-N | 0.835 | 0.015 | | -0.186 | 0.483 | | **1.021** | **0.006** |
| Sit-miR54-N | -0.101 | 0.788 | | -0.160 | 0.664 | | 0.059 | 0.876 |
| Sit-miR55-N | 0.139 | 0.736 | | -0.685 | 0.132 | | 0.824 | 0.091 |
| Sit-miR56-N | 1.077 | 0.110 | | 0.121 | 0.841 | | 0.957 | 0.151 |
| Sit-miR57-N | -0.816 | 0.062 | | -0.180 | 0.624 | | -0.636 | 0.126 |
| Sit-miR58-N | 0.943 | 0.092 | | -0.209 | 0.672 | | 1.151 | 0.053 |
| Sit-miR59-N | **1.253** | **0.001** | | 0.673 | 0.022 | | 0.579 | 0.039 |
| Sit-miR6-N | -0.237 | 0.491 | | 0.097 | 0.773 | | -0.334 | 0.342 |
| Sit-miR60-N | 0.195 | 0.486 | | -0.142 | 0.608 | | 0.337 | 0.246 |
| Sit-miR61-N | -0.315 | 0.384 | | 0.141 | 0.687 | | -0.456 | 0.223 |
| Sit-miR62-N | -0.056 | 0.841 | | -0.090 | 0.743 | | 0.034 | 0.902 |
| Sit-miR64-N | -0.364 | 0.151 | | 0.164 | 0.488 | | -0.528 | 0.054 |
| Sit-miR65-N | 0.181 | 0.647 | | -0.007 | 0.986 | | 0.188 | 0.639 |
| Sit-miR67-N | 1.110 | 0.209 | | -0.067 | 0.935 | | 1.177 | 0.197 |
| Sit-miR69-N | -0.378 | 0.477 | | 0.083 | 0.869 | | -0.461 | 0.394 |
| Sit-miR7-N | 0.224 | 0.519 | | 0.177 | 0.605 | | 0.046 | 0.892 |
| Sit-miR70-N | 1.039 | 0.057 | | 0.667 | 0.173 | | 0.372 | 0.428 |
| Sit-miR71-N | 0.457 | 0.180 | | 0.022 | 0.944 | | 0.435 | 0.202 |
| Sit-miR72-N | -0.079 | 0.783 | | 0.079 | 0.781 | | -0.158 | 0.587 |
| Sit-miR73-N | 0.322 | 0.267 | | 0.144 | 0.602 | | 0.178 | 0.525 |
| Sit-miR74-N | -0.431 | 0.547 | | -0.106 | 0.877 | | -0.324 | 0.650 |
| Sit-miR75-N | 0.193 | 0.576 | | -0.054 | 0.871 | | 0.248 | 0.480 |
| Sit-miR76-N | -0.299 | 0.254 | | -0.283 | 0.275 | | -0.016 | 0.948 |
| Sit-miR77-N | -0.040 | 0.935 | | 0.147 | 0.758 | | -0.187 | 0.709 |
| Sit-miR78-N | -0.816 | 0.062 | | -0.180 | 0.624 | | -0.636 | 0.126 |
| Sit-miR79-N | -0.248 | 0.649 | | -0.405 | 0.444 | | 0.157 | 0.781 |
| Sit-miR8-N | 0.224 | 0.519 | | 0.177 | 0.605 | | 0.046 | 0.892 |
| Sit-miR80-N | -0.705 | 0.416 | | 0.374 | 0.623 | | -1.080 | 0.228 |
| Sit-miR81-N | -0.147 | 0.818 | | 0.265 | 0.652 | | -0.412 | 0.531 |
| Sit-miR82-N | 0.221 | 0.474 | | 0.006 | 0.983 | | 0.214 | 0.489 |
| Sit-miR83-N | -0.470 | 0.249 | | -0.060 | 0.874 | | -0.410 | 0.309 |
| Sit-miR84-N | -0.716 | 0.037 | | -0.149 | 0.596 | | -0.567 | 0.080 |
| Sit-miR85-N | -0.064 | 0.825 | | 0.052 | 0.851 | | -0.116 | 0.689 |
| Sit-miR86-N | -0.503 | 0.258 | | -0.238 | 0.550 | | -0.265 | 0.543 |
| Sit-miR87-N | 0.049 | 0.946 | | 0.324 | 0.643 | | -0.276 | 0.703 |
| Sit-miR88-N | -0.378 | 0.477 | | 0.083 | 0.869 | | -0.461 | 0.394 |
| Sit-miR89-N | -0.618 | 0.476 | | 0.446 | 0.592 | | -1.064 | 0.240 |
| Sit-miR9-N | -0.289 | 0.265 | | -0.282 | 0.273 | | -0.007 | 0.976 |
| Sit-miR90-N | -0.481 | 0.288 | | 0.019 | 0.961 | | -0.500 | 0.277 |
| Sit-miR92-N | -0.125 | 0.728 | | 0.166 | 0.626 | | -0.291 | 0.432 |
| Sit-miR94-N | -0.075 | 0.826 | | 0.320 | 0.350 | | -0.394 | 0.271 |
| Sit-miR95-N | -1.753 | 0.052 | | -0.929 | 0.189 | | -0.824 | 0.323 |
| Sit-miR97-N | -0.021 | 0.943 | | 0.170 | 0.568 | | -0.191 | 0.529 |
| Sit-miR98-N | 0.450 | 0.233 | | -0.240 | 0.505 | | 0.689 | 0.093 |
| Sit-miR99-N | 0.188 | 0.709 | | -0.428 | 0.410 | | 0.616 | 0.258 |
