# Supplementary material for: Integrative RNA sequencing uncovers the miRNA–mRNA landscape of water-deficit response in Setaria italica
Source: Planta. 2026 Aug 10;264(3):92. doi: 10.1007/s00425-026-05117-5 (PMC13457271; doi:10.1007/s00425-026-05117-5)
Supplement: Supplementary file 4 [file 425_2026_5117_MOESM6_ESM.docx]

**Supplementary information for:** Integrative RNA sequencing uncovers a multilayer miRNA–mRNA modules landscape of water deficit response in *Setaria italica*

**Journal:** Planta

**Authors:** M.R. Juliana¹, G.B.T. Vitor¹, G. Gabriel², F.O. Leandro², C. Igor², S. Nathalia¹

**Affiliations:** ¹Centro de Ciências Naturais e Humanas (CCNH), Universidade Federal do ABC (UFABC), São Bernardo do Campo, SP, Brazil. ²LigninLab, Department of Botany, Institute of Biosciences, University of São Paulo, São Paulo, SP, Brazil.

**Corresponding author:** Nathalia de Setta; Email: nathalia.setta@ufabc.edu.br.

**Table S2** Reproducibility across two independent water deficit experiments. The table summarizes the significance of treatment, experiment, and interaction effects based on two-way ANOVA. Significance levels: * *P* ≤ 0.05; ** *P* ≤ 0.01; *** *P* ≤ 0.001; ns, not significant (*P* > 0.05)

| **Trait** | **Treatment effect** | **Experiment effect** | **Interaction** |
| --- | --- | --- | --- |
| Maximum height (cm) | *** (*P* = 4.48e-15) | ns (*P* = 0.2612) | ns (*P* = 0.2186) |
| Total number of leaves | *** (*P* = 1.31e-22) | ns (*P* = 0.1339) | ns (*P* = 0.6332) |
| Shoot biomass (g) | *** (*P* = 4.71e-11) | ns (*P* = 0.3225) | *** (*P* = 1.03e-14) |
| RWC (%) | * (*P* = 0.0396) | *** (*P* = 1e-04) | *** (*P* = 4e-04) |
| Weight of panicle (g) | *** (*P* = 2.76e-05) | ns (*P* = 0.4177) | * (*P* = 0.0325) |
| Panicle length (cm) | *** (*P* = 6.65e-07) | * (*P* = 0.0211) | ns (*P* = 0.1046) |
| Panicle width (cm) | * (*P* = 0.0142) | ns (*P* = 0.0581) | ns (*P* = 0.6687) |
