# Supplementary material for: Integrative RNA sequencing uncovers the miRNA–mRNA landscape of water-deficit response in Setaria italica
Source: Planta. 2026 Aug 10;264(3):92. doi: 10.1007/s00425-026-05117-5 (PMC13457271; doi:10.1007/s00425-026-05117-5)
Supplement: Supplementary file 5 [file 425_2026_5117_MOESM7_ESM.docx]

**Supplementary information for:** Integrative RNA sequencing uncovers a multilayer miRNA–mRNA modules landscape of water deficit response in *Setaria italica*

**Journal:** Planta

**Authors:** M.R. Juliana¹, G.B.T. Vitor¹, G. Gabriel², F.O. Leandro², C. Igor², S. Nathalia¹

**Affiliations:** ¹Centro de Ciências Naturais e Humanas (CCNH), Universidade Federal do ABC (UFABC), São Bernardo do Campo, SP, Brazil. ²LigninLab, Department of Botany, Institute of Biosciences, University of São Paulo, São Paulo, SP, Brazil.

**Corresponding author:** Nathalia de Setta; Email: nathalia.setta@ufabc.edu.br.

**Table S3** Read counts of sRNA-seq libraries before and after filtering and cleaning

| **Library** | **Total reads** | **Clean Reads** | **Reads <18-nt** | **Reads > 25-nt** | **18-nt ≥ reads ≤ 25-nt** |
| --- | --- | --- | --- | --- | --- |
| **100%-1** | 39,389,393 | 39,387,722 | 7,897 | 8,579,706 | 30,800,119 (78%) |
| **100%-2** | 33,437,751 | 33,436,094 | 7,084 | 2,528,647 | 30,900,363 (92%) |
| ***Total-100%*** | 72,827,144 | 72,823,816 | 14,981 | 11,108,353 | 61,700,482 (85%) |
| **30%-1** | 33,247,611 | 33,246,252 | 6,072 | 6,625,405 | 26,614,775 (80%) |
| **30%-2** | 38,240,508 | 38,239,028 | 6,361 | 6,688,972 | 31,543,695 (82%) |
| ***Total-30%*** | 71,488,119 | 71,485,280 | 12,433 | 13,314,377 | 58,158,470 (81%) |
| **0%-1** | 40,448,387 | 40,446,810 | 13,486 | 6,885,610 | 33,547,714 (83%) |
| **0%-2** | 38,235,559 | 38,234,215 | 7,490 | 5,519,189 | 32,707,536 (86%) |
| ***Total-0%*** | 78,683,946 | 78,681,025 | 20,976 | 7,404,799 | 66,255,250 (84%) |
| **TOTAL** | 222,999,209 | 222,990,121 | 48,390 | 31,827,529 | 186,114,202 (83%) |
