# Supplementary material for: Integrative RNA sequencing uncovers the miRNA–mRNA landscape of water-deficit response in Setaria italica
Source: Planta. 2026 Aug 10;264(3):92. doi: 10.1007/s00425-026-05117-5 (PMC13457271; doi:10.1007/s00425-026-05117-5)
Supplement: Supplementary file 6 [file 425_2026_5117_MOESM8_ESM.docx]

**Supplementary information for:** Integrative RNA sequencing uncovers a multilayer miRNA–mRNA modules landscape of water deficit response in *Setaria italica*

**Journal:** Planta

**Authors:** M.R. Juliana¹, G.B.T. Vitor¹, G. Gabriel², F.O. Leandro², C. Igor², S. Nathalia¹

**Affiliations:** ¹Centro de Ciências Naturais e Humanas (CCNH), Universidade Federal do ABC (UFABC), São Bernardo do Campo, SP, Brazil. ²LigninLab, Department of Botany, Institute of Biosciences, University of São Paulo, São Paulo, SP, Brazil.

**Corresponding author:** Nathalia de Setta; Email: nathalia.setta@ufabc.edu.br.

**Table S4** Distribution of clean reads across RNA types (rRNA, tRNA, and unmatched) in the *S. italica* microtranscriptome

| **Library** | **Clean reads** | **%** | **rRNA** | **%** | **tRNA** | **%** | **Unmatched** | **%** |
| --- | --- | --- | --- | --- | --- | --- | --- | --- |
| **100%-1** | 30,800,119 | 100 | 19,482,035 | 63.25 | 321,906 | 1.04 | 10,996,178 | 35.7 |
| **100%-2** | 30,900,363 | 100 | 20,202,925 | 65.38 | 279,763 | 0.90 | 10,417,675 | 33.7 |
| **30%-1** | 26,614,775 | 100 | 18,247,687 | 68.56 | 379,804 | 1.42 | 7,987,284 | 30.0 |
| **30%-2** | 31,543,695 | 100 | 21,139,188 | 67.02 | 401,537 | 1.27 | 10,002,970 | 31.7 |
| **0%-1** | 33,547,714 | 100 | 30,364,986 | 90.51 | 323,488 | 0.96 | 2,859,240 | 8.52 |
| **0%-2** | 32,707,536 | 100 | 21,759,562 | 66.53 | 447,651 | 1.37 | 10,500,323 | 32.1 |
| **TOTAL** | 186,114,202 | 100 | 131,196,383 | 70.49 | 2,154,149 | 1.16 | 52,763,670 | 28.35 |
